# Adaptation of global land use and management intensity to changes in climate and atmospheric carbon dioxide

**DOI:** 10.1111/gcb.14110

**Published:** 2018-03-24

**Authors:** Peter Alexander, Sam Rabin, Peter Anthoni, Roslyn Henry, Thomas A. M. Pugh, Mark D. A. Rounsevell, Almut Arneth

**Affiliations:** ^1^ School of Geosciences University of Edinburgh Edinburgh UK; ^2^ Global Academy of Agriculture and Food Security The Royal (Dick) School of Veterinary Studies University of Edinburgh Midlothian UK; ^3^ Karlsruhe Institute of Technology Institute of Meteorology and Climate Research Atmospheric Environmental Research (IMK‐IFU) Garmisch‐Partenkirchen Germany; ^4^ School of Geography, Earth and Environmental Sciences University of Birmingham Birmingham UK; ^5^ Birmingham Institute of Forest Research University of Birmingham Birmingham UK

**Keywords:** climate change adaptation, CO_2_ fertilisation, food system, land use change, land use intensity, telecoupling

## Abstract

Land use contributes to environmental change, but is also influenced by such changes. Climate and atmospheric carbon dioxide (CO_2_) levels’ changes alter agricultural crop productivity, plant water requirements and irrigation water availability. The global food system needs to respond and adapt to these changes, for example, by altering agricultural practices, including the crop types or intensity of management, or shifting cultivated areas within and between countries. As impacts and associated adaptation responses are spatially specific, understanding the land use adaptation to environmental changes requires crop productivity representations that capture spatial variations. The impact of variation in management practices, including fertiliser and irrigation rates, also needs to be considered. To date, models of global land use have selected agricultural expansion or intensification levels using relatively aggregate spatial representations, typically at a regional level, that are not able to characterise the details of these spatially differentiated responses. Here, we show results from a novel global modelling approach using more detailed biophysically derived yield responses to inputs with greater spatial specificity than previously possible. The approach couples a dynamic global vegetative model (LPJ‐GUESS) with a new land use and food system model (PLUMv2), with results benchmarked against historical land use change from 1970. Land use outcomes to 2100 were explored, suggesting that increased intensity of climate forcing reduces the inputs required for food production, due to the fertilisation and enhanced water use efficiency effects of elevated atmospheric CO_2_ concentrations, but requiring substantial shifts in the global and local patterns of production. The results suggest that adaptation in the global agriculture and food system has substantial capacity to diminish the negative impacts and gain greater benefits from positive outcomes of climate change. Consequently, agricultural expansion and intensification may be lower than found in previous studies where spatial details and processes consideration were more constrained.

## 1 INTRODUCTION

Environmental change will influence future agricultural productivity. Climate impacts have been shown to have both positive and negative impacts on yields depending on crop type and latitude; however, the net global effect of a warming climate on existing cropland is expected to be negative (Deryng, Sacks, Barford, & Ramankutty, 2011; Deryng et al., 2016; Liu et al., 2016; Pugh et al., 2016; Rosenzweig et al., 2014; Tebaldi & Lobell, 2015). Nonetheless, at higher latitudes, increasing temperatures have the potential to increase crop yields (Müller et al., 2015; Pugh et al., 2016). Increased atmospheric levels of carbon dioxide (CO_2_) are also widely expected to increase agricultural productivity, but the magnitude of such CO_2_ fertilisation remains contested (Ainsworth, Leakey, Ort, & Long, 2008; Leakey et al., 2009; Long, Ainsworth, Leakey, Nösberger, & Ort, 2006; Osborne, 2016; van der Kooi, Reich, Löw, De Kok, & Tausz, 2016). Over the coming decades, the food production system will further be affected by increasing demand for agricultural products (Alexander et al., 2015; Foley et al., 2011; Tilman, Balzer, Hill, & Befort, 2011; Weinzettel, Hertwich, Peters, Steen‐Olsen, & Galli, 2013), continued globalisation of trade in agricultural products (D'Odorico, Carr, Laio, Ridolfi, & Vandoni, 2014; Meyfroidt, Lambin, Erb, & Hertel, 2013) and adoption of land‐based climate change mitigation measures (Humpenöder et al., 2015; Smith et al., 2013). However, through shifting land use and changing management practices, the global agriculture and food system can adjust, at least in part, to these changes to lessen the negative impacts and accentuate any potential benefits.

Land use also creates important environmental impacts. For example, 24% of all anthropogenic greenhouse gas emissions (GHGs) in 2010 were associated with agriculture, forestry and other land use (Smith, Bustamante, et al., 2014), and 11% of anthropogenic CO_2_ emissions were associated with land use change (Le Quéré et al., 2016). Expanding agricultural areas and intensifying production—that is, using more inputs, such as fertilisers, pesticides or water or changes in management practices—can increase GHG emissions, deteriorating soil quality, use scarce water and reduce biodiversity (Cassman, 1999; Johnson, Runge, Senauer, Foley, & Polasky, 2014; Newbold et al., 2015; Smith et al., 2013). Furthermore, land‐based mitigation measures may be required to meet current climate change targets (Popp et al., 2017). Understanding how changes in climate, changes in demand for agricultural commodities and land‐based climate change mitigation measures will affect the future agricultural and land use system is therefore critical.

Previous studies have attempted to understand these interactions, using models including a representation of the land use system. Notably, integrated assessment models (IAMs) have been used to investigate climate change mitigation scenarios; for example, considering options such as afforestation, avoided deforestation and bioenergy production (Humpenöder et al., 2014; Popp et al., 2011, 2017; Wise et al., 2009). Under representative concentration pathways (RCPs) (van Vuuren et al., 2011), scenarios with GHG emissions and concentrations that span a range of radiative forcings, and shared socio‐economic pathways (SSPs) (O'Neill et al., 2015), IAMs and other models of land use have projected outcomes (e.g. Calvin et al., 2013; Fujimori, Masui, & Matsuoka, 2012; Havlík et al., 2014; Meiyappan, Dalton, O'Neill, & Jain, 2014; Ren et al., 2016; Stehfest, Vuuren, Bouwman, & Kram, 2014)**.** Interaction between natural drivers, represented by earth system models, and societal drivers, represented in IAMs, have also been undertaken (Collins et al., 2015). Models of the global agricultural system have primarily taken economic equilibrium optimisation approaches, either general (CGE) or partial equilibrium models (Robinson et al., 2014). Due to computational restrictions, these approaches do not typically use high‐spatial resolution when choices regarding rates of agricultural areas and intensities are made, instead representing the globe via a small number of regions or agricultural zones. Crop yields achieved with varying intensities of production are represented using different, but stylised approaches (Nelson et al., 2014). Increases or decreases in agricultural areas are also considered, but with increases specified at regional scales as part of the economic production functions, which are subsequently spatially disaggregated. This assumes that land expansion occurs on progressively less productive land but does not closely relate this expansion to physical properties and limitations. Although downscaling or disaggregating into finer resolution maps is common, nonetheless the optimisation to determine the aggregate land uses within a region (including fertiliser and irrigation rates) has occurred with these aggregate units. An exception to this regional optimisation approach is MAgPIE, which takes a least‐cost optimisation approach using gridded yield data from the global vegetation model LPJml (Lotze‐Campen et al., 2008). However, even in this case, a location‐specific yield response to agricultural input changes is not considered, but rather regional technological change rates are used (Lotze‐Campen et al., 2008). Additionally, MAgPIE aggregates global spatial input data to between 100 and 600 clusters with similar crop yields (Dietrich, Popp, & Lotze‐campen, 2013; Humpenöder et al., 2014; Kreidenweis et al., 2016). Therefore, current global land use models and IAMs do not explore the interactions between agricultural expansion and intensification using crop behaviour from plant‐ecosystem process modelling on a spatially disaggregated basis. Furthermore, to date, there has been a lack of focus in global studies on understanding potential adaptation responses to climate change in land use (Berger & Troost, 2013). IAMs have been widely used to investigate land‐based climate change mitigation options (Humpenöder et al., 2014; Popp et al., 2011, 2017; Rose, 2014; Wise et al., 2009). While most IAMs represent “top‐down” mitigation policies, making the “bottom‐up” nature of the adaptation process more difficult to capture (Hertel & Lobell, 2014).

Here, we present initial results from a novel land use model that uses more detailed biophysically derived yield data and responses to inputs, with greater spatial specificity than previously possible. The approach couples a dynamic global vegetation model (LPJ‐GUESS; Olin, Lindeskog, et al., 2015; Smith, Wärlind, et al., 2014) with a new land use and food system model (PLUMv2). PLUMv2 responds to changes in input (yields and demand for commodities) by endogenously adapting land use at high‐spatial resolution. Greater demand can be met both by intensification and agricultural expansion (Johnson et al., 2014; Tilman et al., 2011). A further novel aspect of this study is that PLUMv2 does not assume market equilibrium, commodity prices are adjusted to account for over‐ or undersupply, while trade mechanisms also allow for representation of international tariffs and transport costs. This offers a more accurate representation of the trade‐offs, responses and cross‐scale interactions that are likely to be important in determining the system dynamics as a whole (Rounsevell et al., 2014). Land use and demand projections from the coupled model system were evaluated against historical data to assess suitability for exploring future scenarios, a task often not conducted for land use models. These coupled models were used to investigate the potential for adaptation to climate change within the agricultural system and possible climate change impacts on land use.

## 2 MATERIALS AND METHODS

### 2.1 Overall coupled model framework

**Figure 1:**
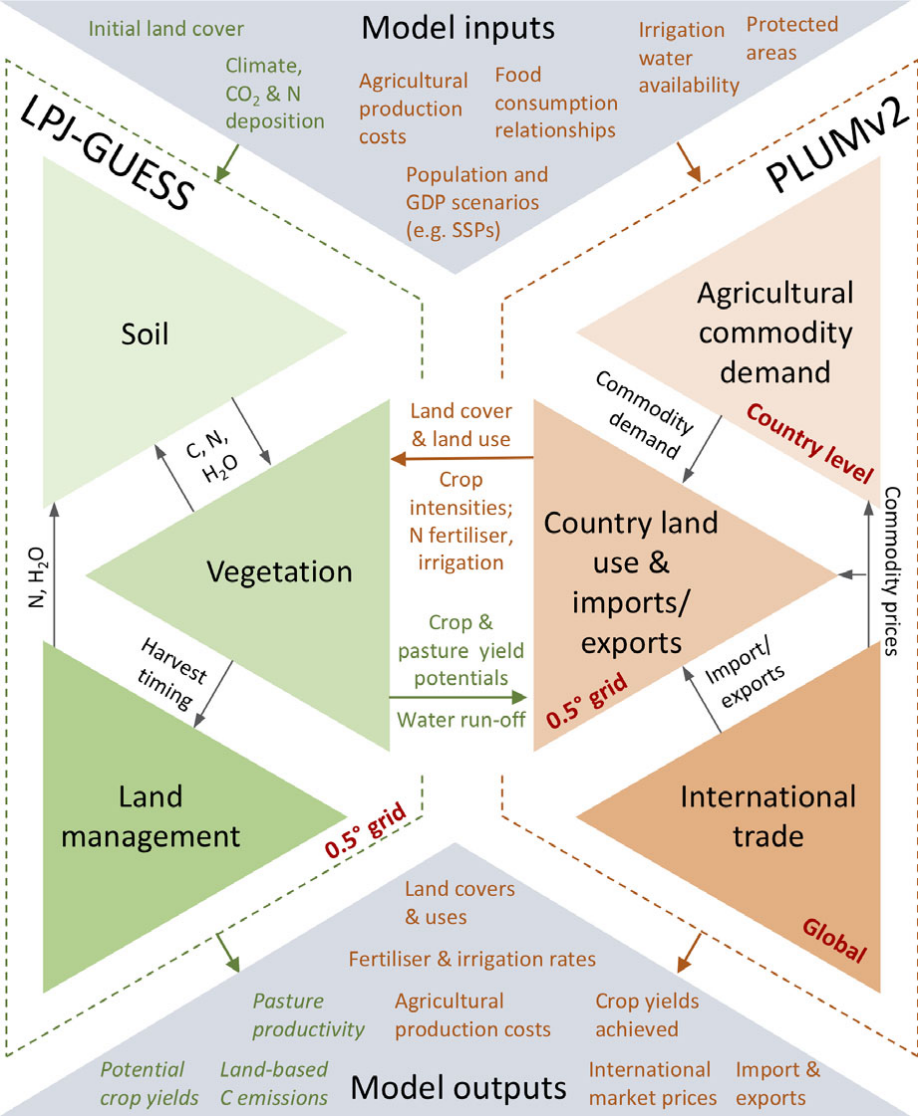
Diagram of main interactions between LPJ‐GUESS and PLUMv2 showing the components and flows within the couple models. Data passed between LPJ‐GUESS and PLUMv2 are on a 0.5° grid. Water run‐off is aggregated to food production units (FPUs), and adjusted for other uses, before being used to constrain water use in PLUMv2. Both models run at annual time steps, with LPJ‐GUESS output data averaged over a 5‐year period for input into PLUMv2 (see Figure S1 for temporal interaction details) [Colour figure can be viewed at http://wileyonlinelibrary.com]

The Lund–Potsdam–Jena General Ecosystem Simulator (LPJ‐GUESS; Smith, Wärlind, et al., 2014) global vegetation model was coupled to PLUMv2, a new and reconceptualised version of the Parsimonious Land Use Model (Engström, Rounsevell, et al., 2016). LPJ‐GUESS produced crop and pasture yield potentials on a 0.5° grid—using a factorial experiment for crops with three fertilisation rates and rain‐fed vs. irrigated conditions—using climate forcing scenarios. PLUMv2 used these yields in combination with scenario data, for example GDP and population, to project land use and management inputs (Figure 1). The components of the coupled model are discussed further below.

### 2.2 Biophysical crop yield potentials across intensities from LPJ‐GUESS

LPJ‐GUESS uses a dynamic global vegetation model approach to simulate terrestrial ecosystems and their interactions with large‐scale biogeochemical processes. It combines a mechanistic representation of physiological processes for a number of broad vegetation categories (plant functional types) with population dynamics based on forest gap modelling to simulate plant growth, death, competition and succession (Hickler et al., 2004; Smith, Prentice, & Sykes, 2001; Smith, Wärlind, et al., 2014). Cropland and pasture were represented in LPJ‐GUESS as fractions of land distinct from “natural” vegetation that undergo management and harvest (Lindeskog et al., 2013). Pasture is simulated as a natural grassland but with the addition of an annual grazing “harvest” term. Analogously to natural vegetation, the wide variety of crops planted around the world is simplified into several crop functional types (CFTs). Each CFT was assigned parameters related to plant physiology (e.g. photosynthetic pathway and vernalisation requirements) and management (e.g. fertilisation regime). The LPJ‐GUESS crop model includes nitrogen cycling and has been shown to realistically simulate yield responses to nitrogen and CO_2_ fertilisation (Olin, Schurgers, et al., 2015).

LPJ‐GUESS was used with four CFTs: winter‐sown C3 cereals (TeWW), spring‐sown C3 cereals (TeSW), C4 cereals (TeCo) and rice (TrRi) (Olin, Lindeskog, et al., 2015). LPJ‐GUESS input data and parameterisation details are available in the supporting information, along with information on changes made to crop water demand, soil moisture and irrigation. Potential yields under six alternative combinations of fertiliser and irrigation rates were determined. Three rates of fertilisation were considered: zero fertiliser, 200 and 1,000 kgN/ha, with each either rain‐fed or fully irrigated (i.e. with as much water applied as the plants would take up), with the potential heat units scheme for plant development (Olin, Lindeskog, et al., 2015). The high‐fertilisation rate is substantially beyond that used in practice, but represents a maximum upper limit of achievable yields. Economic considerations are accounted for in the land use optimisation and act to limit the fertiliser modelled as applied.

#### 2.2.1 Calibration to observed crop yields

PLUMv2 used seven crop types to represent demand for agricultural products, mapped on to the four LPJ‐GUESS CFTs (Table 1), with the aim of maintaining realistic physiological and management parameters. A calibration routine was used to translate yields produced by LPJ‐GUESS into potential yields for each PLUMv2 crop type, for example, TeSW to pulses. The calibration process was also used to improve the fidelity of LPJ‐GUESS yields to observations for crops it was designed to simulate (e.g. TrRi to observed rice yields). The calibration factors were generated via a slope‐only regression between simulated and observed per‐area yields for the years 1995–2005 (Table 1). Observed yields for each PLUMv2 crop type were derived from FAO data (FAOSTAT, 2015a, 2015b), except for energy crops, data for which were taken from the Biofuel Ecophysiological Traits and Yields Database (BETYdb, LeBauer et al., 2010). Figure S2 shows the scatter plots comparing the simulated and observed yields for each PLUMv2 crop type, and Table 1 gives the derived calibration factors. The yields used by PLUMv2 were calculated as the product of the calibration factors and associated CFT yield output from LPJ‐GUESS.

**Table 1:** Mapping between crop and consumption types used in FAOStat, LPJ‐GUESS and PLUMv2. LPJ‐GUESS crop functional types are TeSW for spring C3 cereals, TeWW for winter C3 cereals, TeCo for C4 cereals and TrRi for rice

PLUMv2 crop type	FAOSTAT (2015b) crop types	LPJ‐GUESS crop type	Calibration factor from LPJ‐GUESS to PLUMv2
Cereals C3	Wheat Barley Oats	Higher of TeSW or TeWW for each grid cell	0.988
Cereals C4	Maize Millet Sorghum	TeCo	0.706
Rice	Rice paddy	TrRi	0.978
Oil crops	Oil crops primary	Higher of TeSW or TeWW for each grid cell	0.594
Pulses	Pulses total	TeSW	0.572
Starchy roots	Roots and tubers total	TeSW	5.832
Energy crops	Miscanthus^a^	TeCo	2.148

a Data on Miscanthus come from the Biofuel Ecophysiological Traits and Yields Database (LeBauer et al., 2010).

#### 2.2.2 Yield potentials in the land use model

The yields available for any combination of fertiliser and irrigation rate were estimated using the calibrated yield potentials at alternate irrigation and fertilisation rates for each grid cell and crop. An exponential yield function for all types of intensity was used that fits the LPJ‐GUESS yield potentials provided (see supporting information—Methods for full equations). As well as fertiliser and irrigation rates, the level of management practices was represented by a “management intensity,” encompassing activities such as pesticide application rates, reseeding of grassland, controlling of soil pH, for example, through application of lime, and larger stock of machinery or labour. An exponential approach was also used to represent diminishing returns from increasing management inputs. Yield increases from technology change, for example, due to plant breeding, were included as an annual exogenous increment to these yields.

**Figure 2:**
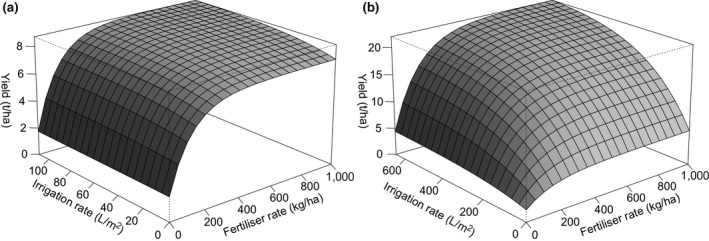
Example yield responses to fertiliser and irrigation inputs at 2010, for spring wheat (a) in Aberdeenshire, Scotland (lat: 57°, lon: −2.5°), and (b) maize in Texas, USA (lat: 30°, lon: −96°), at maximum management intensity

Examples of the surfaces produced for two grid cells and crops are shown in Figure 2. Figure 2b shows a location with a high response to irrigation rates, in comparison with Figure 2a, and both cases demonstrate a response with diminishing returns from increasing fertiliser.

### 2.3 Land use and agricultural trade model from PLUMv2

#### 2.3.1 Agricultural commodities demand

Demand for agricultural commodities was projected at a country level for six commodity groups—cereals, oil crops, pulses, starchy roots, ruminant products and monogastric products—considering both food and bioenergy requirements. The proportions of commodities in each group were fixed from the baseline year of 2010, and cereal demand could be met by wheat, maize or rice.

Food demand was projected based on log‐linear relationships with per capita income. Country‐level historical data on GDP, population and consumption from 1961 to 2010 (FAOSTAT, 2015a, 2015c) were used to derive these relationships, with data points weighted by country population. Projections used GDP and populations from the SSP scenarios (O'Neill et al., 2014). Dietary patterns alter as incomes change, with higher incomes being associated with a shift from staples such as starchy roots and pulses, to commodities such as meat, milk and refined sugars (Fiala, 2008; Kearney, 2010; Keyzer, Merbis, Pavel, & van Wesenbeeck, 2005; Tilman et al., 2011; Weinzettel et al., 2013). However, further increases in income tend to lead to lower increases in the rate of consumption (Cole & McCoskey, 2013), while the consumption of the less preferred product, for example, pulses, drops but at a decreasing rate. Both of these observations can be accounted for by the approach, similar to that applied by Tilman and Clark (2014) and Bodirsky et al. (2015); however here, the approach is applied to multiple commodity groups rather than to calorific intake and aggregate animal product consumption.

Cultural and other variations between countries lead to differences between (a) the consumption implied by the regression relationship from population and income and (b) the observed consumption in the same year. For example, Japan has less meat consumption than the global relationship suggests given its per capita income, but with a high level of fish consumption. The difference between expected and observed consumption rates for each country was calculated in the baseline year of 2010. Under some scenarios, these differences were held constant; under others, an exponential convergence was applied to global dietary patterns as per capita GDP increased (see supporting information—Methods). The historical and projected consumptions plotted against GDP are shown in Figure S3.

First‐ and second‐generation bioenergy demand trajectories were specified exogenously to represent a moderate business‐as‐usual scenario. Bioenergy demand for food commodities‐that is, first‐generation bioenergy‐were modelled from an observed baseline level of demand (Alexander et al., 2015; FAOSTAT, 2015c) adjusted to double by 2030 from the 2010 level and thereafter remain constant. Demand for dedicated energy crops (i.e. second‐generation bioenergy) was specified as a global trajectory that increases to 4,000 Mt DM/year by 2100 from 34 Mt DM/year in 2010, in line with the SSP2 demand with baseline assumptions (Popp et al., 2017). Demand for second‐generation bioenergy was not associated with individual countries, with all production locations determined endogenously.

#### 2.3.2 Country‐level optimisation of land use, livestock production and international trade

For each country and time step, the agricultural land use and level of imports or exports were determined through a least‐cost optimisation that meets the national demands for food commodities. For example, an increased national demand for a commodity can be met in three ways—increasing the land area for growing associated crops; increasing the levels of inputs to achieve higher yields, that is, intensification; or increasing the level of net imports, that is, reducing exports or increasing imports. The land use and intensities are spatial (0.5° grid), while the imports and exports rates are national. Costs were associated with each aspect, using prices in 2010 US$. The model constraints, equations and the objective function are given in the supporting information—Method. The 47 countries with a population of more than 25 million in 2010 were represented separately, and countries with small populations were aggregated regionally using the six World Bank regions (World Bank, 2014). Livestock nutrition requirements were calculated using feed conversion ratios (Alexander, Brown, Rounsevell, Finnigan, & Arneth, 2016). Monogastric livestock was considered to only consume feed, while ruminant livestock nutritional requirements could be met from a mix of pasture and feed, providing the opportunity for intensification by increased feed rates and a substitution between pasture and cropland.

Agricultural land use costs per unit area were calculated from a global base crop cost plus a cost of each of the three inputs considered, that is, fertilisation, irrigation and management intensity. The base costs are a minimum cost to producing that crop. The input costs were all products of the intensity rate and a cost rate. The base crop cost was estimated from a third of the cost per hectare in an intensive production system costs (Alexander & Moran, 2013; SAC Consulting, 2013), assuming reductions in inputs (e.g. seed rate, agrochemical and machinery use) could save cost, but with the implications for yields achieved (yield potentials in the land use model section above). To obtain the maximum possible yields—those output by LPJ‐GUESS for a given fertiliser and irrigation rate—the remainder of costs associated with these current intensive production practices were included in the management intensity costs plus additional cost for higher agrochemical usage or machinery use. The base costs for pasture were assumed to be low, representing extensive grazing; intensive systems would include substantial management costs, for example, to represent reseeding to improve pasture yields. The crop costs parameters used are given in Table S2. An index of irrigation costs per unit of water varied spatially (Figure S4) based on an aridity index (CGIAR‐CSI, 2008). The required irrigation rate for each crop and grid cell to minimise plant water stress was calculated in LPJ‐GUESS, allowing the simulated water usage and implied cost to be determined in PLUMv2. The water use efficiency of irrigation—the ratio of irrigation water requirements to the water withdrawn (FAO, 2017)—was taken as 0.5, within the 0.294–0.855 range of irrigation efficiencies globally (Rost et al., 2008). For each grid cell, the total irrigation water used across crops was constrained by water availability. Each year, the water available for irrigation was determined from the LPJ‐GUESS‐simulated runoff, assuming water consumption by sectors other than agriculture following Elliott et al. (2014). Runoff aggregated into food production units (FPUs; Kummu, Ward, de Moel, & Varis, 2010) was adjusted to account for domestic and industrial water uses, environmental limitations on water extraction and to reproduce Elliott et al. (2014) in a baseline year of 2010. Future water consumption for non‐agricultural sectors used SSP2 projections (Elliott et al., 2014). The water remaining per FPU was allocated equally across the grid cells within each FPU to determine the irrigation water available.

Costs arising from changes in land cover—between natural and agricultural land or between cropland and pasture—were calculated per area converted (Table S3). The conversion of natural land cover to agricultural land was restricted by protected areas from the World Database on Protected Areas (WDPA; IUCN & UNEP‐WCMC, 2015). Terrestrial protected areas with a WDPA status of “established,” specified on a 0.5° grid, were prevented from being converted to agricultural use. China's National Forest Protection Program was implemented as an annual limit to deforestation of 1.1% in these areas (Ren et al., 2015). A minimum natural area fraction was applied to preserve at least a proportion of forest or other natural land cover within each location, where protected areas did not meet this threshold. Expansion of agricultural areas was taken equally from forest and other natural vegetation. Urban and barren (e.g. ice‐covered) land areas were constant from LUH2 in 2010 and not available for agricultural land expansion.

The final country‐level cost relates to imported and exported agricultural commodities. Within the model, a single global market tariff‐free price exists for each commodity and time period. The revenue received for exports was accounted for at this international market price. However, prices of imported commodities were inflated to account for transportation costs, losses during transportation and import trade tariffs (Anderson, Martin, & Valenzuela, 2006). The net import levels were initialised from observed values (FAOSTAT, 2015a, 2015c).

#### 2.3.3 Global trade balance and prices

In PLUMv2, as in reality, supply and demand in the global market for each commodity need not be in equilibrium, where over‐ or undersupply for commodities are buffered through stock variations (FAOSTAT, 2015c). The modelled international market prices for each commodity were adjusted exponentially using market conditions to provide a feedback mechanism (Ghoulmie, Cont, & Nadal, 2005). For example, where larger quantities of a commodity are exported globally than imported, the price for that commodity decreases; this reduces the benefits from its export and reduces the cost of importing it, creating a tendency to correct for the oversupply. The initial prices for each commodity were set exogenously (Index Mundi, 2016) but subsequently adjusted endogenously from the rate of under‐ or oversupply in the market (see supporting information). Global stocks for each commodity accommodate periods of over‐ and undersupply and were explicitly modelled. Initial stock levels were derived from FAO Commodity Balance data (FAOSTAT, 2015a, 2015c) following the method of Laio, Ridolfi, and D'Odorico (2016).

#### 2.3.4 Spin‐up and spatial clustering in PLUMv2

PLUMv2 was initialised with GDPs, populations, net imports and demand from FAOSTAT (2015a, 2015c), and land covers from Land Use Harmonisation version 2 (LUH2; Hurtt, Chini, Frolking, & Sahajpal, 2017), at 2010. Net imports were constrained to be equal to the FAOSTAT net imports. The aim was to obtain land uses, including intensities, that generate the observed country‐level commodity production and are close to the LUH2 land covers, in the initialisation year. Intensity data (i.e. for fertiliser, irrigation or management input levels), were not provided to the initialisation process. To ensure that modelled land use changes occur only because of future scenario shifts, the initialisation was run iteratively, for the same demand and net import rates, but using the land use results from the previous iteration. This process was continued until a stable solution (<0.4% change in area or intensity values between iterations) was reached (around 10 iterations).

Optimising land use decisions on a 0.5° grid involves the computationally challenging task of finding a solution to the non‐linear optimisation problem for potentially large numbers of locations; China's 942 Mha of land, for example, is represented by around 3,800 0.5° grid cells. To reduce computational requirements while retaining spatial accuracy, similar but potentially non‐contiguous grid cells within a country were grouped. Mean crop yields and land cover type areas were calculated for each cluster, and optimisation occurred at the cluster level. The land use changes indicated by the optimisation results were then mapped back to the original grid cells in proportion to the available natural area. A *K*‐mean clustering approach (Macqueen, 1967) was used with randomly initialised centroid clusters for each country. The resulting number of clusters in each country was dependent on the size and homogeneity, for example, 176 in USA and 140 in Russia. The approach is similar to that in the MAgPIE land use model, which uses between 100 and 600 clusters globally, divided across 10 regions (Dietrich et al., 2013; Humpenöder et al., 2014; Kreidenweis et al., 2016). PLUMv2 here used around 3,400 clusters globally, with a mean cluster size of 3.5 Mha. Therefore, the PLUMv2 model for each year and ensemble member had around 100,000 decision variables across the country optimisation, as for every cluster, there were four decision variables (i.e. area, fertiliser, irrigation and other intensity) for each of the eight land use types (seven for crop types plus pasture). There were also decision variables at a country level for each commodity: for example, for livestock feed usage and import and export quantities; however, the total number of the country‐level variables was small compared to the number of spatial variables. More clusters were used than in MAgPIE due to the country‐level approach in PLUMv2 and the desire to represent spatial heterogeneity within countries.

### 2.4 Benchmarking to historical data

#### 2.4.1 Demand benchmarking

To test the demand projection approach, the FAOSTAT (2015a, 2015c) data were divided into a time series for calibration (1961–1990) and a time series for benchmarking (1990–2010). The demand regression relationships were derived from the calibration data as described above. These relationships were used from 1990 to project demand for each country to 2010 given the historical population and GDP data (World Bank, 2014). Countries that split into multiple states after 1990, for example, the USSR, were handled as the post‐1990 separate states, and the earlier combined historical socioeconomic data disaggregated by population.

**Figure 3:**
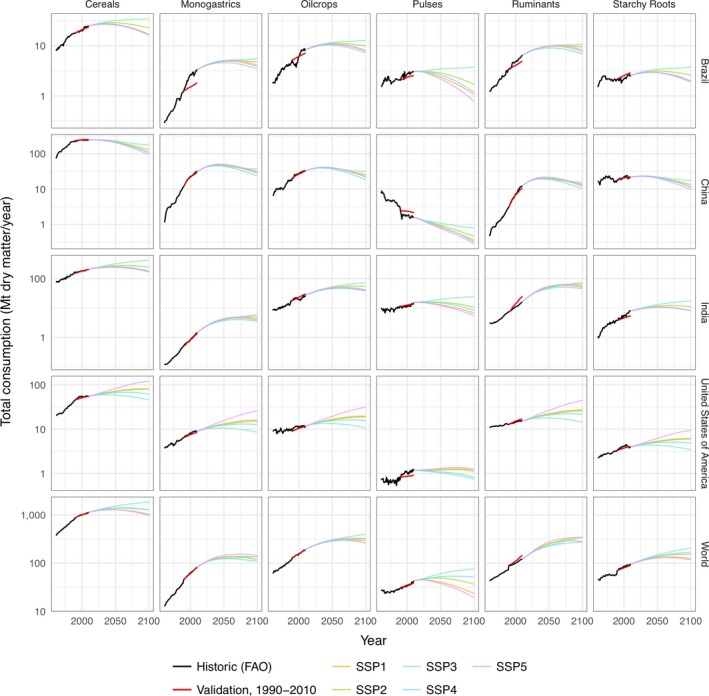
Historic and projected demand for the modelled agricultural commodity groups. The FAO data (black lines) show historic demand from 1961 to 2011 (FAOSTAT, 2015a, 2015c). The benchmarking data (red lines) use 1961–1990 FAO data to calibration relationships and 1990–2010 GDP and population (World Bank, 2014) to project demand in that period. Projections of demand under each SSP scenario from a 2010 baseline (other coloured lines), using 1961–2010 FAO data set for calibration and the OECD socio‐economic scenario data (IIASA, 2014) [Colour figure can be viewed at http://wileyonlinelibrary.com]

A comparison of the projected consumption for the period 1990–2010 against the observed FAO consumption values (FAOSTAT, 2015a, 2015c) showed similar patterns of change for global and country‐level demand (Figure 3). At 2010, the largest global percentage difference was seen in ruminants, a commodity group in which demand increased by 60% globally between 1990 and 2010, with the projections 15% higher than the FAO values (FAOSTAT, 2015a, 2015c). Monogastric livestock was the only commodity with a larger growth, increasing by 78%, but here, the PLUMv2 projections were 1% lower globally in 2010 than the FAO value. This may indicate a shift in animal product preference from ruminant products to monogastrics between the time periods of the split data sets. Nonetheless, the modest level of these differences and the ability to reproduce the patterns of country and global changes in demand for the validation period suggest that the demand projection approach is adequate for the purposes of the land use modelling exercise being conducted. However, one limitation is that the approach assumes a continuation of the relationship between income and food demand and therefore does not account for potential future changes or transformation in food preferences (Alexander, Brown, et al., 2017; Stehfest et al., 2009).

#### 2.4.2 Land use benchmarking

The land use results were benchmarked by initialising the model at 1970 and running to 2010, then comparing the 2010 model results against historical estimates at 2010 of cropland and pasture areas as well as fertiliser and irrigation use. LUH2 at 1970 (Hurtt, Chini, Frolking, et al., 2017; Hurtt, Chini, Sahajpal, & Frolking, 2017) was used to initialise land covers, with yield data taken from the LPJ‐GUESS benchmarking runs (see supporting information). Demand data from 1961 to 2010 (FAOSTAT, 2015a, 2015c) were used to derive the demand relationships. This does not provide an independent verification of the demand projections, but such a test has already been completed (as outlined above). Comparing land use changes from 1990 was considered to provide an insufficiently long time series, compounded by the relatively low land use changes from that date.

**Figure 4:**
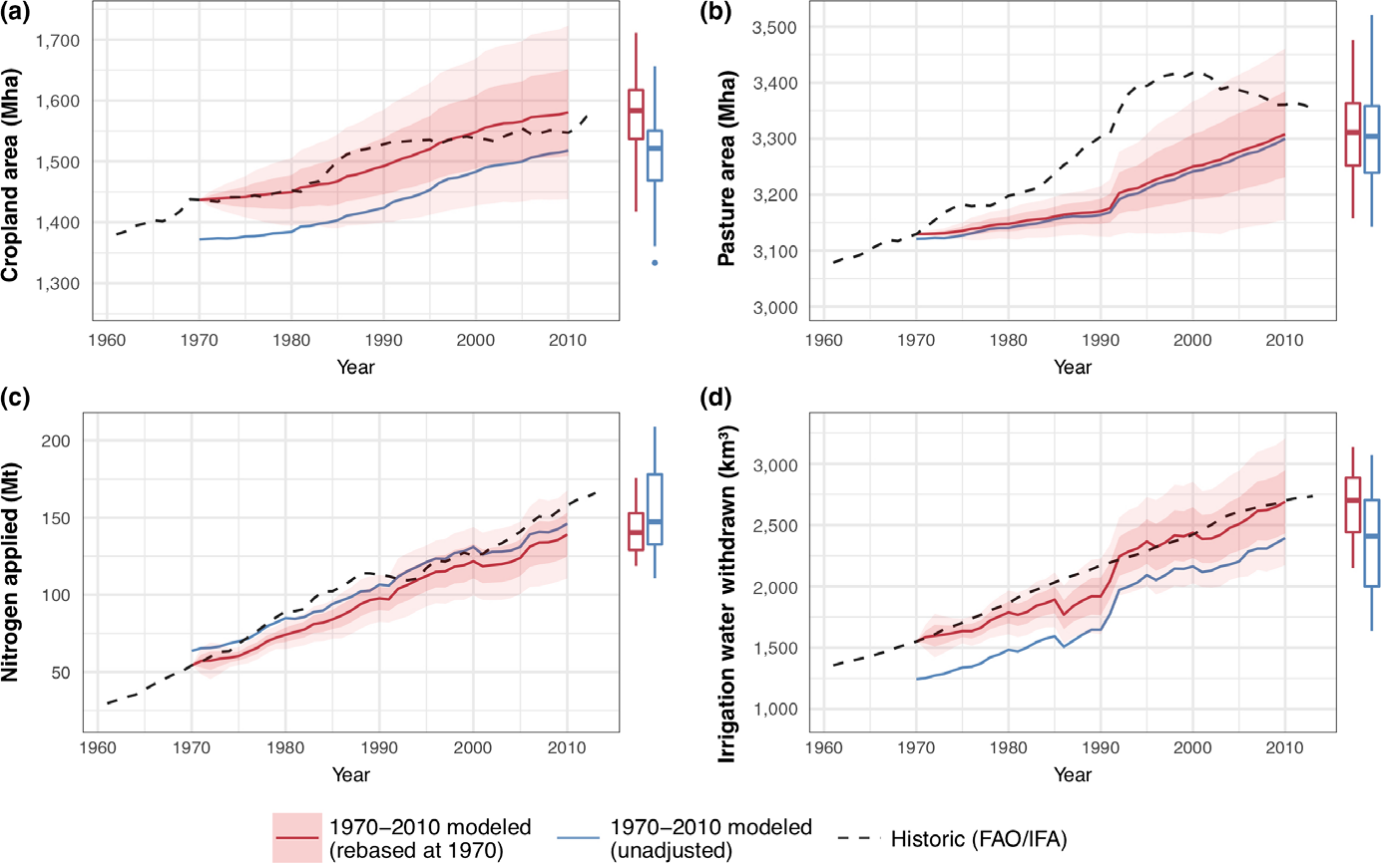
Global comparison of historic (FAO/IFA) agricultural land use data against benchmark LPJ‐GUESS/PLUMv2 simulation, for (a) cropland and (b) pasture area, and (c) nitrogen and (d) irrigation water used on cropland. Values are plotted both unadjusted, and with simulated results rebased to the historic values at 1970 to show changes from that date more clearly. Uncertainty ranges were determined using a stochastic sampling method (n = 50), with shaded areas showing one and two standard deviations around the median. Box plots are for the modelled values at 2010, showing median, interquartile range, up to 1.5 interquartile range whiskers, and outliers [Colour figure can be viewed at http://wileyonlinelibrary.com]

The modelled and FAOSTAT (2015d) global cropland and pasture areas from 1970 to 2010 are shown in Figure 4a–b. Nitrogen (N) and irrigation water applied was also compared to historical estimates (Figure 4c–d). Historical N use was estimated from the world inorganic fertiliser use (IFA, 2017) plus N applied to cropland from manure. The 43.3 Mt of N applied to cropland from manure in 2000 (Bouwman, Boumans, & Batjes, 2002) was scaled by the livestock production index (FAOSTAT, 2017) to give a time series of historical manure N rates. Historical irrigation water extracted was estimated from global irrigation water extraction of 2,700 km^3^ in 2010 (AQUASTAT, 2016), scaled by the irrigated cropland area (FAOSTAT, 2015d) for other years. The level of uncertainty arising from definitional differences and data acquisition issues is unknown (Prestele et al., 2016).

**Figure 5:**
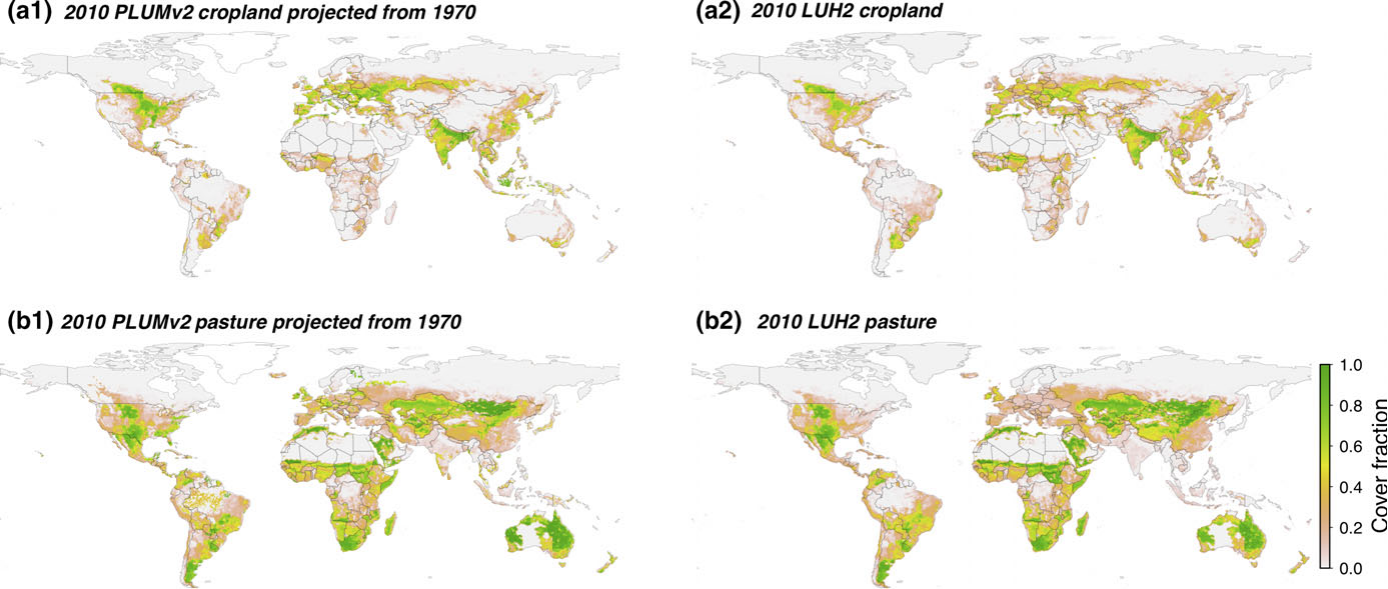
Cropland and pasture land cover fractions in 2010 (a1 & b1), from PLUMv2 benchmarking projections with 1970 baseline and (a2 & b2), from LUH2 (Hurtt, Chini, Frolking, et al., 2017) [Colour figure can be viewed at http://wileyonlinelibrary.com]

The impact of parameter uncertainty on the historical model results was tested using a stochastic approach. Uniform distributions of model parameters (Table S3) were sampled over a range of 50% above and below the central parameter values using a Sobol sequence method with *n* = 50 (Chalaby, Dutang, Savicky, & Wuertz, 2015). The median and standard deviation of global cropland area, pasture area and nitrogen and irrigation water used were calculated for each year (Figure 4). Figure 5 shows the distribution of cropland and pasture land covers from the benchmarking process using the central parameter values compared to the widely used LUH2 data set (Hurtt, Chini, Frolking, et al., 2017; Hurtt, Chini, Sahajpal, et al., 2017).

The benchmarking results (Figure 4) demonstrate that the model reproduces a net global expansion and intensification in the period 1970–2010 with a reasonable degree of accuracy. For example, cropland expanded by approximately 8% and fertiliser use increased around threefold in both the model and historic data. The median‐unadjusted cropland areas are around 50 Mha lower than the FAO data, as PLUMv2 is not constrained to reproduce the baseline land cover in the initial year. Total irrigation water use was also slightly lower than historical estimates, but again tracked the changes over time. To adjust for these offsets, model values rebased to the historical values at 1970 are shown in Figure 4. As a percentage of the absolute value, the uncertainty of fertiliser and irrigation was greater than that for cropland or pasture areas. For example, the interquartile range for cropland was 5% of the 2010 value, while for fertiliser use, it was 17% and 31%, respectively, for the rebased and unadjusted values. Perhaps the greatest discrepancy in the global aggregate comparison was that for pasture area changes, which suggests that the model's projections are biased towards underprediction of pasture change. This is potentially due to the high diversity and associated complexity encompassed by this land cover (including problems in defining what is considered pasture) and is consistent with higher uncertainty seem for pasture area projections from other models (Alexander, Prestele, et al., 2017; Prestele et al., 2016).

The cropland and pasture distributions in 2010 (Figure 5) demonstrate a high correspondence to the results of LUH2. Historical estimates of global land use and land cover—including LUH2—are model outputs typically calculated using a combination of primary sources such as satellite data and country‐level statistics (Klein Goldewijk, Beusen, Van Drecht, & De Vos, 2011; Klein Goldewijk et al., 2010; Monfreda, Ramankutty, & Foley, 2008; Ramankutty, Evan, Monfreda, & Foley, 2008). As such, estimates of historical global land distributions for the same date vary between different models, for example, LUH2 vs. SAGE (Ramankutty et al., 2008) and differences between PLUMv2 results and LUH2 could result from uncertainty in either model. Geographic differences between PLUMv2 and LUH2 include PLUMv2's output of a lower cropland area in sub‐Saharan Africa and South America but greater cropland area in China. Processes that are not modelled may give rise to inaccuracies in the PLUMv2 results; for example, Chinese policies and direct involvement in some sub‐Saharan African and South American countries (Cotula, Vermeulen, Leonard, & Keeley, 2009; Zoomers, 2010) may have suppressed domestic expansion in China, displacing it to other countries. In China, cropland expansion in PLUMv2 was concentrated in south‐eastern regions, corresponding closely to where forest loss has been observed (Ren et al., 2015)—a behaviour not replicated in LUH2. The PLUMv2 results also show some pasture (~5 Mha) in northern latitudes, for example, Finland, that is not in LUH2 (Figure 5). This may be due to yields of pasture from LPJ‐GUESS being higher than obtainable in these areas or because PLUMv2 takes no account of current accessibility or proximity to existing populations or infrastructure. Another potential reason for differences is incomplete or inaccurate protected area information, for example, where all protection policies are not represented within the protected areas database used (IUCN & UNEP‐WCMC, 2015), or protections are not fully enforced. These reasons may also contribute to the difference in pasture areas in the Amazon, where greater pasture expansion was seen than in LUH2.

Given the agreement between agricultural expansion and intensity with the historical estimates as well as the concurrence of spatial distributions, the modelling was considered appropriate for exploration of future scenarios.

### 2.5 Scenario descriptions

The aim of the scenario design was to explore the adaptation of the land use system to a range of climate and CO_2_ forcings to 2100, using the RCPs (van Vuuren et al., 2011). The same “middle of the road” socio‐economic scenario—SSP2 (O'Neill et al., 2014, 2015)—was used for all scenarios to investigate the impact of climate, without the complexity and potentially offsetting or exacerbating impact of other scenario changes. The scenarios used should not be taken as equally likely, as different combinations of SSP and RCP are not equally plausible; for example, the probability of RCP2.6 and SSP2 is low (Engström, Olin, et al., 2016). Furthermore, by only varying climate and CO_2_ forcing, the scenarios are not intended to represent a full range of plausible future states.

The adaptation of land use was simulated under four RCPs, which represent differing intensities of climate change and future atmospheric CO_2_ concentrations. A further experiment (“constant‐climate + CO_2_”) was performed by repeatedly using the 1981–2010 climate with detrended temperature and atmospheric CO_2_ levels and nitrogen deposition rates from 2010. This produces temperatures and precipitation with interannual variability, but using constant climate, CO_2_ and nitrogen deposition. Population and GDP trajectories were taken from SSP2 (O'Neill et al., 2014, 2015) using World Bank projections (IIASA, 2014). Bioenergy trajectories were assumed without large‐scale land‐based climate mitigation, with global demand for dedicated second generation bioenergy crops increasing to 4,000 Mt/year by 2100 (Popp et al., 2017). PLUMv2 model parameter uncertainty was included in the projections, using the stochastic uncertainty approach described for the benchmarking process. Further details of LPJ‐GUESS and PLUMv2 inputs can be found in the supporting information—Methods.

## 3 RESULTS

**Figure 6:**
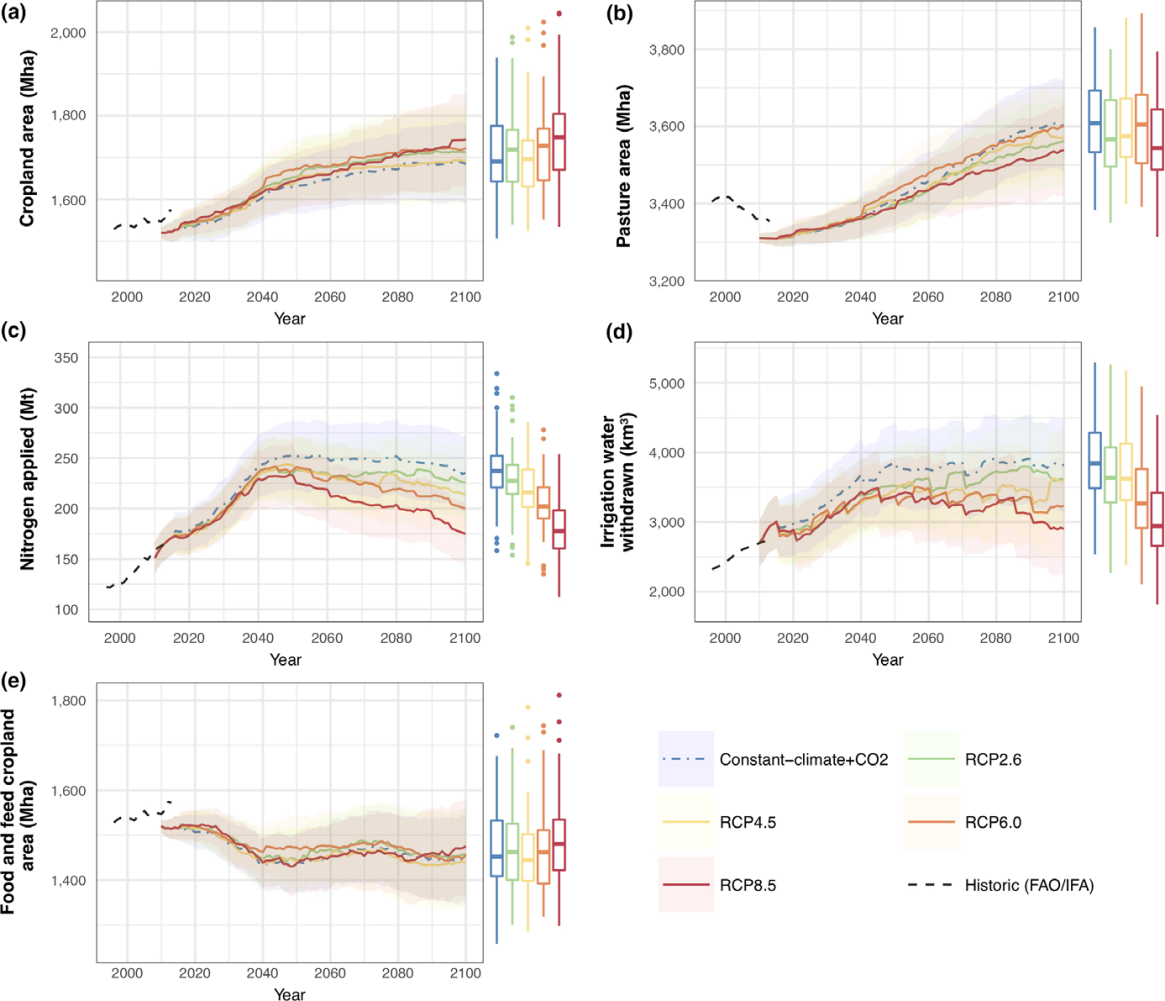
Global agricultural land use results from 2010 to 2100 under RCP climate scenarios and constant climate. Other scenario parameters were identical in all simulations, with socio‐economic values from SSP2 and baseline bioenergy adoption. Uncertainty ranges for each RCP were determined using a stochastic sampling method of model parameters (n = 50). Box plot distributions for 2100 values are shown, as per Figure 4

Figure 6 shows global cropland and pasture areas from 2010 to 2100 resulting under each of the climate forcing scenarios. Total median cropland increases to between 1,690 and 1,743 Mha, an increase of 170–223 Mha. These cropland areas include areas of dedicated second‐generation energy crops, which expanded to 242–262 Mha by 2100. Total cropland expansion was less than the energy crop area increase, and therefore cropland for food and feed decreased (by 45–82 Mha). Median pasture increased by 291–3,601 Mha with RCP6.0 and by 228–3,538 Mha with RCP8.5. In all scenarios, the historic growth of nitrogen fertiliser application rate continued until about 2040–2050. For example, in RCP2.6 fertiliser use increases from 151 Mt in 2010 to a peak of 241 Mt in 2045 before reducing slightly to 225 Mt in 2100; in RCP8.5 fertiliser application declines more substantially to 175 Mt by 2100, from a peak in 2049 of 234 Mt. The trend for irrigation water use followed some similar patterns to that of fertiliser, with strong growth until 2040–2050 before either declining (e.g. RCP8.5) or remaining relatively stable (e.g. RCP2.6). The global area‐weighted mean yields achieved, excluding energy crops, increased from 3.0 t/ha to around 4.4–4.6 t/ha by around 2040 in all full RCPs, after which the rate of increase decreased, with yields in the range 4.5–4.8 t/ha until 2100. The Constant climate + CO_2_ simulations resulted in the lowest median cropland area at 2100 (1,685 Mha) and the highest of pasture (3,603 Mha), although the differences in area between this experiment and the RCP results were small (29 Mha less cropland and 42 Mha more pasture than RCP 2.6). Constant climate + CO_2_ required more nitrogen and irrigation water than any of the RCP scenarios, with 235 Mt of nitrogen and 3,820 km^3^ of irrigation water withdrawn in the median result at 2100 (10 Mt more nitrogen and 210 km^3^ of water than RCP 2.6).

A pattern of lower fertiliser and irrigation use at 2100 with more intense climate change scenarios is apparent, although the effect was small in magnitude compared to the uncertainty in results from the range of model parameters tested (Figure 6). For example, the interquartile range for nitrogen application in 2100 was 211–240 Mt with RCP2.6 and 155–194 Mt with RCP8.5. The results from the Constant climate + CO_2_ scenario also suggest that greater increases in fertiliser and irrigation use are required, in comparison to the climate change scenarios. The distributions of cropland areas, both including and excluding energy crop areas, from the simulations of each scenario are skewed towards higher values, that is, outlier results have high cropland areas, with a similar uncertainty for each climate forcing.

**Figure 7:**
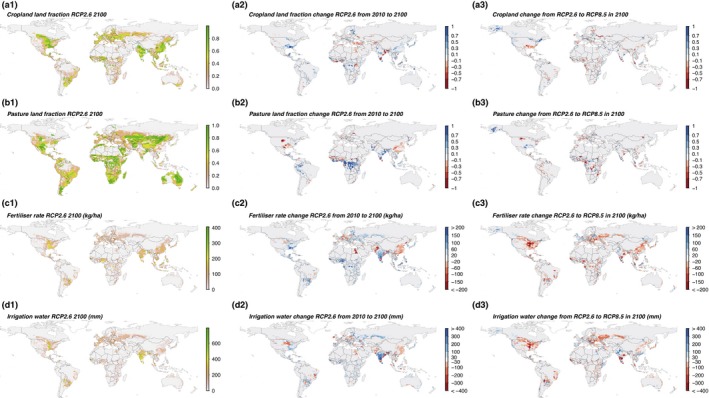
Maps of land use in 2100 under RCP2.6, change from 2010, difference between RCP2.6 and RCP8.5 values in 2100, using SSP2 and central parameter values. Land use values shown are cropland and pasture land cover fractions (a & b), and crop fertiliser and irrigations rates (c & d) [Colour figure can be viewed at http://wileyonlinelibrary.com]

In all scenarios, the modelled geographic distributions of land cover changes between 2010 and 2100 show a combination of both agricultural land abandonment and expansion as well as substitution between cropland and pasture (Figure 7). Increases and decreases in fertiliser and irrigation inputs were also suggested to occur at different locations. Some of these input changes are associated with the change in cropland areas, for example, reductions in fertiliser and irrigation linked with abandonment of cropland, such as in Egypt—which sees a corresponding increased dependence on imports. This is an example of a wider trend in these results, where increasing globalisation in the food system shifts production to the areas where costs of production are low. As a result, the percentage of modelled global demand met from international imports increased from 12% to 25% between 2010 and 2100 in a central RCP4.5 scenario. The pattern of agricultural input rates generally decrease in North America and northern Europe, while increasing in Africa and South America.

The largest changes in land covers are the areas of pasture expansion in the Congo basin seen in all the climate scenarios. For example, in the median parameter RCP2.6 case, 120 Mha around the Congo were converted to pasture by 2100 and as well as 20 Mha in the Amazon Basin (Figure 7‐b2).

The diversity of response both within and between countries can be seen by comparing the results of RCP2.6 and RCP8.5 in 2100 (Figure 7a3‐d3). For example, in RCP8.5 relative to RCP2.6, Brazil, China and the USA all increase output from pasture, reduce costs of pasture production and reduce reliance on feed in livestock production, due to increases in pasture yield potentials from the higher climate forcing intensity. However, other outcomes from these countries differ. Pasture in the USA increases by a smaller area (25 Mha) in 2100 for RCP8.5 compared to RCP2.6, while decreasing in Brazil by 10 Mha and remaining relatively constant in China (although with some shifts in location). Production from cropland in the USA decreases overall, with the difference being met by a lower use of livestock feed (food demand is the same in all scenarios and net exports rates remained relatively constant), supported by higher pasture productivity, due to the climate and CO_2_ differences. Cropland areas in China and Brazil decrease marginally (around 5 Mha), but increase in the USA by a more substantial 30 Mha. The total level of intensity measured by cost of production decreases between these RCP results at 2100, for example, for wheat drop of 19% in China and 23% in the USA, and a smaller decrease for maize of 9% in China and 1% in the USA. Changes in cropland in the USA could be characterised as agricultural production extensification, with increasing area and lower inputs. However, there is a substantial shift in location of agriculture and balance of crops grown, for example, cropland abandonment in the southeastern USA (some of which convert to pasture), and an expansion of cropland in more northern states, including Alaska. Under lower climate forcing scenarios, the expansion of cropland into Alaska is not seen. The cropland expansion is used to primarily for wheat production, which the abandoned areas were previously primarily maize. This is associated with a switch from maize to wheat of around 40 Mha in the USA. Similar patterns are seen globally, with global maize decreasing by 20% and wheat increasing by 42%.

## 4 DISCUSSION

### 4.1 Comparisons to previous land use projections

Previous studies projecting land cover areas have found a wide range of cropland and pasture areas (Alexander, Prestele, et al., 2017) encompassing the results produced here. These previous projections, however, include many scenarios that do not correspond to those tested. Under similar socio‐economic conditions, that is, SSP2, cropland area for food across five IAMs expanded by 50–350 Mha (Popp et al., 2017), while having broadly similar bioenergy demand and area to the PLUMv2 results. Contrastingly, the PLUMv2 results have a reduction of 45–82 Mha across the RCPs tested here. Pasture from the five IAMs range from −200 to +250 Mha, with their “marker” model indicating an increase in 250 Mha, close to the median PLUMv2 cases of 228–293 Mha. Perhaps, the most direct comparison to previous scenarios is with Constant climate + CO_2_ scenario here with the SSP2 “baseline” scenario from Popp et al. (2017). The results found here have a median change of −74 Mha cropland for food and feed, +238 Mha for energy crop and +293 Mha pasture area, while the Popp et al. (2017) marker model results are +200 Mha for food and feed, +200 Mha for energy crops and +250 Mha pasture area. The difference in energy crop areas may be a result of different demand; in 2100, Popp et al. (2017) assumed 3,500 t/year dry matter, whereas this study used 4,000 t/year. However, the differences between these model outcomes are small in comparison with the uncertainty ranges in either study. Details of fertiliser and irrigation rates have not typically been reported in detail in other land use model studies, making comparison difficult. However, the PLUMv2 results show a continuation of currently observed trends until around 2045, implying an increase in global nitrogen application of 90 Mt (60% increase in current total) and 800 km^3^ additional water extraction (30% increase) by that date. The more constant or declining inputs after around 2045 coincide with the slowing in SSP2 of both global population rise and income‐driven dietary transitions to substantially reduce the rate of food demand increases (Figure 3).

The lower cropland expansion found by the coupling of LPJ‐GUESS and PLUMv2, compared to some previous studies, may be due to our detailed yield response representation. This allows both input levels and land use to be varied by the model based on biophysically derived yield responses. The model can therefore identify efficient approaches to fulfil demand as changes occur, for example, to climate, market conditions or demand. Abandonment and expansion of agricultural land or increases and decreases in production intensity may all occur within the same country and time. For example, modelled irrigation water usage rates will change in response to water availability, plant water requirements, crop yield potentials and demand. Perhaps, most straightforwardly, an increase in demand could be met by increasing water inputs. However, irrigation rates change due to variation in water availability due to climatic change. Plant water requirements are also responsive to climate conditions as well as atmospheric CO_2_ concentrations and rates of nitrogen fertilisation, leading to changing irrigation demands (Figure 2). Similarly, nitrogen fertilisation rates are influenced by a range of factors operating at local, country and global scales.

### 4.2 Protected areas

The model includes a representation of protected areas, where conversion into agricultural land was not permitted (see Method section for details). Nonetheless, expansion of agricultural areas occurred in locations of global importance for biodiversity and climate regulation, including the Amazon and Congo basins. The agricultural expansion occurred in locations not currently specified as protected in the IUCN, UNEP‐WCMC (2015) database of protected areas used. Such a land use change would likely have major environmental impacts, including biodiversity loss and climate effects at local and global scales (Bala et al., 2007; Gibson et al., 2011; Malhi et al., 2008). Possible future policies for avoiding deforestation or existing policies that provide a level of non‐spatially specific protection are not contained in this protected area database, and therefore are not included in these results. For example, economic approaches that incentivise a reduction in deforestation (Bustamante et al., 2014) were not represented. If such polices were included, deforestation and conversion into pasture or cropland may be reduced in the associated areas. If land use change was to be avoided in this way, other consequences in the model would arise through the indirect effects caused by the displacement of production from areas no longer entering agricultural use (Popp, Humpenoeder, et al., 2014). The indirect effects could include the expansion of cropland and pasture in other less protected areas, increases in intensity on existing agricultural land or most likely a combination of both. Similar adjustments in results could occur if the cost of conversion from forest to agricultural land cover were increased, with a greater conversion of other land covers to agriculture combined with higher intensity of production. Greater competition for land could also arise from climate mitigation policies, for example, supporting bioenergy use or afforestation to provide a terrestrial carbon sink (Albanito et al., 2016; Popp, Rose, et al., 2014). Investigating land use outcomes and displacement effects under climate mitigation policies were out of scope for this study, but could be addressed in future work.

### 4.3 Role of adaptation and impacts of climate change on agriculture

Adaptation of land use decisions provides a mechanism to moderate the potential impact of climate change, or changes in demand, on the global agricultural and food system. The greater the change in climate and the more substantial the impact on crop yield potentials (Figure S5), the more likely new opportunities will be created to take advantage of beneficial changes or to mitigate the impact of negative shifts, for example, by changing crop types, management practices or agricultural locations. These climate changes might be relatively localised and differentially impact locations within regions or countries. The LPJ‐GUESS and PLUMv2 coupling provides a method to incorporate climatic changes and to investigate the adaptive global land use system responses.

At a global aggregate scale, the pattern of increased intensity of climate forcing (including increased atmospheric CO_2_ level) is linked to higher yield potentials, reduced nitrogen losses and greater water use efficiency, which leads to lower fertiliser and irrigation inputs in the PLUMv2 results (Figure 6c,d). Reduced nitrogen losses under elevated CO_2_ occurred in the model because faster crop growth rates allow more fertiliser to be taken up by the plant before it is lost via leaching or gas emission. The apparently counter‐intuitive relationship between intensity of agricultural production and climate forcing comes about because of the fertilising and water‐saving impacts of increased atmospheric CO_2_ levels, combined with the possibility for the global land use system to adapt to minimise cropland area in those regions negatively affected by climate change and maximise cropland area in those where potential is increased. The result is a relatively complex pattern of global cropland area change (Figure 7). Pasture area is influenced by the pasture and cropland yield potentials and cropland area changes. Increases in crop yield potentials encourage greater use of feed in animal production by reducing the associated production cost. Cropland and pasture area changes interact due to the costs of land cover conversion. Consequently, for example, areas where cropland is abandoned are often converted to pasture: for example, in west Africa, India and the USA (Figure 7). Overall, notwithstanding some variations, higher intensity of climate forcing appears to lead to larger cropland expansion and less pasture expansion.

Rates of agricultural expansion would be reduced if we assume higher costs of agricultural expansion or other policies to protect forest or other non‐agricultural land covers. The current parameter range tested produces benchmarking results in the 1970–2010 period in line with other available data sets. However, associated costs may have changed over time, and current and future expansion of agricultural land may be more constrained and costly than during the historic period. Further scenario development, for example, using the SSP framework, and uncertainty analysis would help understand this more fully, but was out of scope for the analysis presented here. Our results assume technology change in plant breeding, which provides an annual increase in yield above that which can be achieved by increasing intensification (the central parameter value used was 0.2%, Table S3). Higher rates of technology improvements—which could be achieved by, for example, the introduction of genetically modified or gene edited organisms—would reduce the expansion of agricultural land or inputs. Conversely, if technology improvements were not able to achieve these gains, then more land and other inputs to agricultural production would result.

### 4.4 Limitations of the approach

PLUMv2 is not constrained to reproduce initial land covers used in the calibration process. Imposing such a constraint could lead to rapid changes in initial simulation years. Therefore, the approach of finding a stable state in proximity to a calibration data set, comprising land covers as well as national production, consumption and international trade data, was preferred. No data on fertiliser or irrigation use were provided to the model calibration, in part due to a lack of suitable available data, and therefore, the initial fertiliser and irrigation rates were derived endogenously during the calibration process. The PLUMv2 simulation calibration outcomes in 2010 are close to the historical estimates (Figure 6), making this potential difference of minor importance in the future simulation results. There are greater differences in the benchmarking runs starting in 1970 for cropland and irrigation water use (Figure 4). For example, cropland in 1970 was 65 Mha (5%) lower, and irrigation water use 300 km^3^ (20%) lower, than historical estimates (FAOSTAT, 2017; IFA, 2017), although the high uncertainty in these estimates complicates any benchmarking. Although the benchmarking process produced a reasonable fit to observed aggregate global outcomes and land cover distribution from LUH2, discrepancies were noted. The explanations suggested above for these differences—for example, influence on land use change in proximity to existing infrastructure, imperfect protected area enforcement, and effects of bilateral trade agreements between countries—could be implemented to test the outcome from altering these assumptions.

The demand projections assume a continuation of historical income–demand relationships and thus do not consider possible alterations in dietary preferences, for example, towards lower meat consumption for both health and sustainability reasons (Stehfest et al., 2009). Furthermore, there was no price elasticity of demand, and so the types and quantities of commodities demanded do not alter in response to price changes, but only population and per capita incomes. Given the objective to investigate adaptation in response to alternative climate futures, we believe such assumptions are acceptable. However, to investigate other scenarios, for example, which include dietary trend adjustments, other assumptions and approaches would be required.

Soil degradation—including from erosion, compaction, sealing and salinisation (Smith et al., 2016)—was not included in the modelling conducted. Agricultural land lost to degradation between 2000 and 2030 was projected to be 30–87 Mha (Lambin & Meyfroidt, 2011), with 7.5% of grassland degraded because of overgrazing (Conant, 2012), while erosion degradation can lead to compensatory benefit at the site of deposition (Lal, 2001). Changes in soil pH resulting from excessive nitrogen fertilisation were also not considered. Continued land degradation increases the pressure on land, but is perhaps smaller in magnitude than other drivers considered, for example, socio‐economic and climate changes. Nonetheless, it would be advantageous to include the effect of soil degradation within models such as LPJ‐GUESS and PLUMv2.

## 5 FUTURE RESEARCH

This study applied newly coupled models to study the response to climate changes for a single fixed socio‐economic scenario. Further work is required to explore the response to alternative socio‐economic conditions, for example, using the SSPs, and to a range of potential climate change mitigation measures, for example, bioenergy and measures to reduce deforestation and increase afforestation. There are also important aspects of crop response to climate change, such as heat stress and CO_2_ fertilisation, which are currently the subject of high uncertainty and merit further investigation. A key aim of the coupled LPJ‐GUESS and PLUMv2 modelled framework was to allow the feedback for land use change on climate as well as the climate impacts on land use, to be considered. Further work is planned to continue model development and to integrate these feedbacks, using a climate emulator (IMOGEN; Huntingford et al., 2010), to study the response in a fully couple climate, vegetation and land use modelled system.

The results suggest that the global agriculture and food system has the capacity to potentially diminish the negative impacts and take greater advantage of the more positive outcomes of climate change through adaptation, for example, by changing crop types, management practices or shifting cultivated area. These adaptations are spatially specific, given geographic variability in climate change impacts on agricultural production. Outputs from models projecting future land uses without accounting for detailed spatial‐, crop‐ and input‐specific factors may therefore be biased towards overestimating land use impacts under a changing climate. To quantify this potential bias, further work is required to establish the extent modelled land adaptation is affected by the level of detail in the representation of spatial and input factors. The results found here suggest that increased intensity of climate forcing reduces the inputs required for food production, largely due to the fertilising and enhanced water use efficiency effects of elevated atmospheric CO_2_ concentrations. However, achieving this requires substantial shifts in the global patterns of intensity of production, with greater inputs required in Africa and South America, and reductions in North America and Western Europe. Such changes in land use and management intensity have consequences for other ecosystem services, and thus, the apparent resilience in the food system indicated by this study may lead to degradation of other ecosystems.
