# Supplementary material for: Adaptation of global land use and management intensity to changes in climate and atmospheric carbon dioxide
Source: Glob Chang Biol. 2018 Mar 24;24(7):2791–809. doi: 10.1111/gcb.14110 (PMC6032878; doi:10.1111/gcb.14110)
Supplement: Supplementary file 1 [file GCB-24-2791-s001.pdf]

Supplementary Information for Adaptation of global land use and management intensity to changes in climate and atmospheric carbon dioxide

SI Materials and Methods

Model interaction details

Three groups of runs were performed: the “calibration,” “benchmarking,” and “future” runs.

The calibration run was used to provide simulated yield over 1995–2005 for the purposes of calibrating LPJ-GUESS crop yields to yields from the FAO data (Section 2.2.1). After a 500-year spin-up, a transient run—using historically-varying climate, atmospheric CO_2 concentration, and land use (see section “LPJ-GUESS input data and parameters” for details)—began in 1901 and ended in 2005.

The benchmarking run, spanning 1901–2010, was used for the benchmarking procedure described in Section 3.2. As with the calibration run, the benchmarking runs began with a 500-year spin-up followed by a transient period—the “initial phase”—which ended in 1960. At this point, the benchmarking run split into two parallel simulation tracks (Figure SI-1). The “actual” track continued to use historical land use data, starting with 1961 and saving the model state every five years (end of 1965, end of 1970, etc.). The “potential” track consisted of a series of 10-year runs using a dummy land use dataset where each grid cell contained some pasture and some area of each crop×irrigation×fertilizer treatment. This track was designed to provide input to PLUM regarding the potential performance of each crop, management input level, and pasture from one five-year period, for the purposes of planning land use and management inputs in the next five-year period. “Potential” runs began with the average soil state from the preceding year’s “actual” state file; to allow for equilibration of crop phenological parameters, the first five years of each “potential” run were discarded. Thus, for example, the state from the end of the 1971–1975 “actual” run would be used to

initialize the "potential" run for 1976–1985, which would in turn provide performance data for 1981–1985 to PLUM, which would be used to determine land use and management for 1986–1990. The period 1961–2010 is referred to as the "alternating phase" of the benchmarking runs.

The future runs, spanning 1850–2100, began as the calibration and benchmarking runs did—with an initial spin-up followed by a transient phase using historically-varying climate, CO_2 concentration, and land use. This initial phase lasted from 1850–2000, after which the runs split into "actual" and "potential" tracks using the same basic structure as the benchmarking run's alternating phase (Figure SI-1).

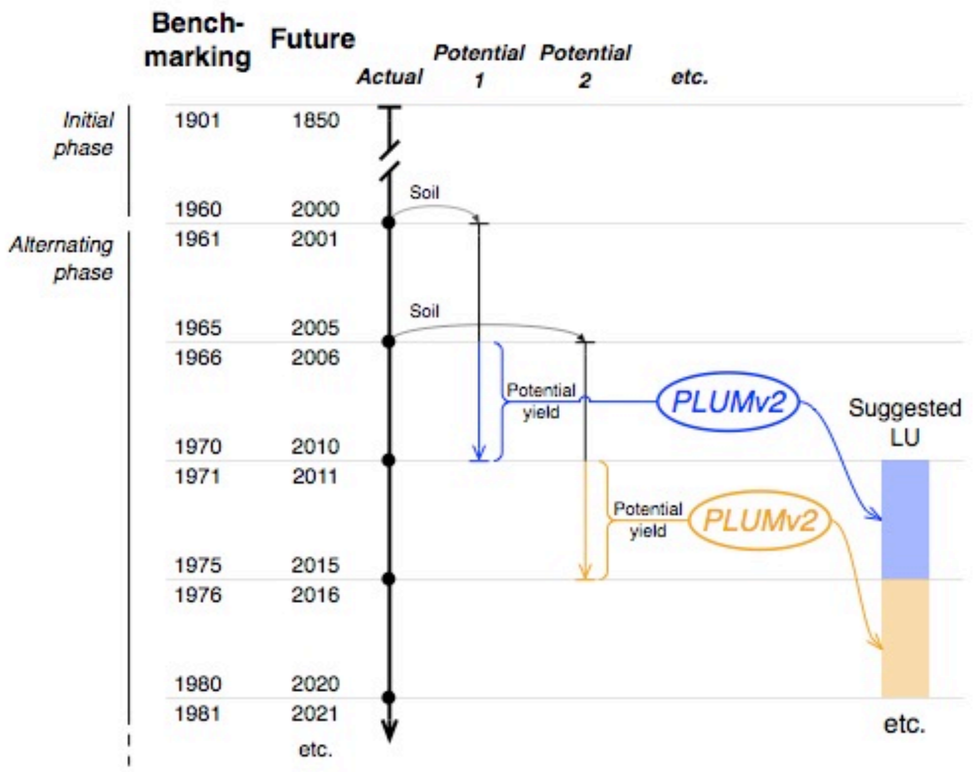

Figure SI-1. Sequence diagram of the benchmarking and future runs in LPJ-GUESS and interactions with PLUMv2.

However, whereas there was just one actual and one potential track in the benchmarking run's alternating phase, there were 16 of each for the future runs. Four runs used climate and CO_2 projections for each of the four Representative Concentration Pathways (RCPs)—RCP2.6, RCP4.5, RCP6.0, and RCP8.5—which denote increasingly intense scenarios of climate change. A constant-

climate+CO_2 experiment was also included (see Section 4). In all future "actual" runs, land use distribution was held constant after 2010.

LPJ-GUESS input data and parameters

In the calibration and benchmarking runs, LPJ-GUESS was forced with 1901–2010 climate data from CRU-NCEP version 7 (Le Quéré *et al.*, 2016), but using CRU TS3.24 precipitation (Harris *et al.*, 2014) due to problems discovered in the CRU-NCEP precipitation data. The future runs used climate forcings and atmospheric CO_2 from the 1850–2100 IPSL-CM5A-MR projections from the Fifth Coupled Model Intercomparison Project (CMIP5; (Taylor *et al.*, 2012)). The IPSL-CM5A-MR forcings were bias-corrected (Ahlström *et al.*, 2012) to the 1961-1990 observation-based climate used by the calibration and benchmarking runs.

During the calibration run, as well as the initial phases and actual tracks of the benchmarking and future runs, the fractions of cropland and pasture in each grid cell were taken from the Land Use Harmonization version 2 (Hurtt *et al.*, 2017). The fraction of cropland in each of eight CFT×irrigation categories (four CFTs, either irrigated or rainfed) was determined based on the MIRCA2000 dataset (Portmann *et al.*, 2010), mapped to the LPJ-GUESS CFTs as described in Table SI-1. In the initial phase and actual tracks of the benchmarking and future runs, no irrigation was applied. Nitrogen fertilizer application rates for each crop type were based on the AgMIP_NUTRIENTS.HARM.version1.0 dataset prepared for the Global Gridded Crop Model Intercomparison exercise (Elliott *et al.*, 2015) of the Agricultural Model Intercomparison Project (AgMIP), and were also assumed not to vary with time.

The runs were spun up for 500 years. The spin-up for the calibration and benchmarking runs used repeated 1901–1930 climate, 1901 CO_2 mixing ratio (296.1 ppmv), and 1901 land use; the future runs' spin-up used repeated 1850–1879 climate, 1850 CO_2 mixing ratio (284.7 ppmv), and 1850 land use.

Table SI-1: Mapping used for crop area mixture during calibration.

LPJ-GUESS CFT	MIRCA2000 crop(s)
TeWW / TeSW	Wheat, barley, rye
TeSW	Cassava, groundnuts/peanuts, pulses, potatoes, rapeseed (canola), sugarbeet, sunflower, soybeans
TeCo	Maize, millet, sorghum
TrRi	Rice

LPJ-GUESS changes

Previously, on irrigated fields, LPJ-GUESS only applied enough water to leave soil moisture at the wilting point (i.e., no more plant-available water) after plant uptake. Whilst sufficient to capture the effects of irrigation on the modelled plant, this method did not allow for a realistic calculation of irrigation water consumption (AQUASTAT, 2016). We changed the irrigation behaviour; LPJ-GUESS now applies enough water so that soil moisture on irrigated fields does not drop below a certain threshold even after plant uptake, evaporation, percolation, and runoff. These thresholds vary based on LPJ-GUESS crop type: 45% and 47.5% of plant-available water for wheat and maize respectively, and 80% of total soil water for rice (after depletion fractions listed in Table 22 (Allen *et al.*, 1998)). We also changed the order of operations so that irrigation demand was calculated *after* precipitation and snowmelt input to the soil, and to allow irrigation to trigger percolation.

Changes were also made to the calculations of water demand and supply for crop plants (see Sitch *et al.* (2003) for relation of demand and supply to plant water stress). The calculation of water supply (i.e., the amount of water that a plant could take up given current soil conditions and transpirative capacity) has been reworked. Previously, water supply was a linear function of modelled phenological stage (i.e., the current leaf area index divided by the maximum leaf area index, LAI, observed in the previous growing season). Water demand, on the other hand, is not a linear function of phenological stage. This sometimes resulted in water demand exceeding water supply even in moist soils and with evapotranspirative demand below the 5 mm day^{-1} maximum plant transpiration rate (Sitch *et al.*, 2003). We replaced the direct linear dependence of crop plants' water supply on phenological stage

with a linear dependence on the ratio of wdemand (today's water demand) to wdemand_leafon (what wdemand would be if today's plants had the highest LAI recently observed for this CFT in this grid cell). Maximum observed crop LAI was updated daily instead of at the end of each year, to ensure that the wdemand ≤ wdemand_leafon.

We also added a soil moisture depletion factor, Ks, that was multiplied with water supply for crop plants. This variable represents how soil moisture extraction becomes more difficult as the soil gets drier. When soil moisture was above a crop's irrigation threshold, Ks = 1; below that threshold, Ks reduces linearly until reaching 0 at available soil water = 0 (Allen *et al.*, 1998).

LPJ-GUESS yield calibrations

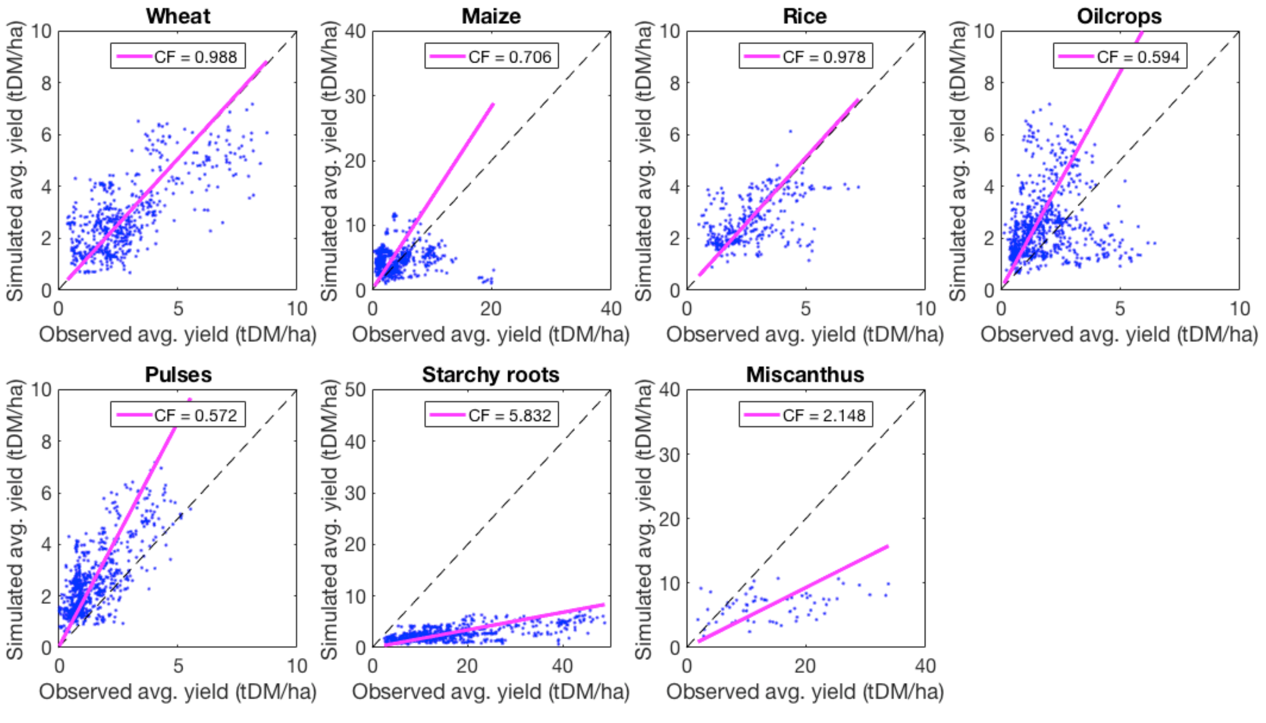

Figure SI-2. Observed to LPJ-GUESS yield scatter plots, with regression line used to determine calibration factors.

Yield response

The yield y for a given location, crop, year and climate were calculated as a function of fertiliser rate f, irrigation rate w, and management intensity m, at time t, as:

$$yield(f, w, m, t) = \left(A + B.(1 - \mathrm{e}^{-\alpha.f}) + C.(1 - e^{-\beta.w}) + D.(1 - \mathrm{e}^{-\alpha.f}).(1 - e^{-\beta.w})\right)$$
$$* (1 - e^{-\gamma.m}) \; * \; (1 + \delta(t - t_0))$$

where α, β, γ, A, B, C and D are all scalar parameters, specific to that grid cell location, crop, year and climate. The intensities f, w and m are indices from 0 to 1, where 0 is no or the minimum input and 1 the maximum. For example, irrigation intensity of 0 represents rain-fed, while 1 represents sufficient irrigation to avoid plant water stress. Parameters α, β and γ control the rate of increase in yield for the three intensities and how quickly returns diminish, while the A, B, C and D control the yields achieved. δ is the annual technology change rate, specified in exogenously in the scenario, and t_0 the baseline time-point.

For each location, crop, year and climate, the parameters in the above equation were calculated from LPJ-GUESS output that provides yield potentials at different levels of irrigation and nitrogen fertilisation. The parameters selection ensured that the yield surface would perfectly fit the zero- and mid-points of irrigation and fertilisation, and asymptotically approach the yield at maximum water and nitrogen application. No yields at a mid-rate of irrigation were available and therefore an 80% increase in yield from 50% of the irrigation rates was assumed. The management intensity parameter γ also uses this rate of increase and was therefore set to 3.22 (i.e. approximately -ln(1-0.8)/0.5).

$$A = y_t(f_{min}, w_{min})$$

$$B = y_t(f_{max}, w_{min}) - y_t(f_{min}, w_{min})$$

$$C = y_t(f_{min}, w_{max}) - y_t(f_{min}, w_{min})$$

$$D = y_t(f_{max}, w_{max}) + y_t(f_{min}, w_{min}) - y_t(f_{min}, w_{max}) - y_t(f_{max}, w_{min})$$

$$\alpha = -ln\left(1 - \frac{y_t(f_{mid}, w_{min}) - y_t(f_{min}, w_{min})}{y_t(f_{max}, w_{min}) - y_t(f_{min}, w_{min})}\right) * \left(\frac{f_{min} - f_{max}}{f_{mid} - f_{min}}\right)$$

$$\beta = -ln\left(1 - \frac{y_t(f_{min}, w_{mid}) - y_t(f_{min}, w_{min})}{y_t(f_{min}, w_{max}) - y_t(f_{min}, w_{min})}\right) * \left(\frac{w_{max} - w_{min}}{w_{min} - w_{mid}}\right)$$

where $y_t(f_{min}, w_{min})$ is the yield potential (from LPJ-GUESS) at time t, given minimum fertilisation, irrigation and management input. f_{max} and w_{max} are fertilisation and the irrigation rates that maximises theoretical yield, f_{mid} and w_{mid} are intermediate rates of fertilisation and irrigation, between f_{min} *and* w_{min}, and f_{max} and w_{max}. γ is the rate parameter to management intensity response, m, and δ is the scenario technology change rate, applied between time t, and t_0. Crop yields in PLUMv2 are adjusted based on calibration factors from best fit regression to FAO yields (FAOSTAT, 2015), which converts the yields into wet matter terms. Pasture yields are handled as dry matter throughout (Alexander *et al.*, 2016).

Demand projections

The demand per capita for a commodity was given as:

$$demand_{pc} = \frac{f(I_t)}{f(I_{t0})}.(observed_{t0}.\Delta - f(I_{t0}).(1 - \Delta))$$

$$\Delta = e^{z.(I_t - I_{t0})/I_{t0}} \quad and \quad z = \frac{\ln(assumed\_closure\_rate)}{gdp\_rate\_change}$$

where *f(x)* is the best-fit log-linear regression function of demand for that commodity at an income per capita, x. I_t and I_{t0} are the incomes per capita, respectively, at time t, and the baseline $t0$. $observed_{t0}$ is the commodity demand in the baseline year. z is the dietary closure parameter, with 0 indicating no convergence in global diets. The z parameters were determined from a scenario assumption of the rate of closure to global dietary patterns given an increase in GDP. However, in all model scenarios presented z was set to zero, i.e. no closure of dietary differences. This set Δ=1 and the demand per capita to:

$$demand_{pc} = \frac{f(I_t)}{f(I_{t0})}.\left(observed_{t0} - f(I_{t0})\right)$$

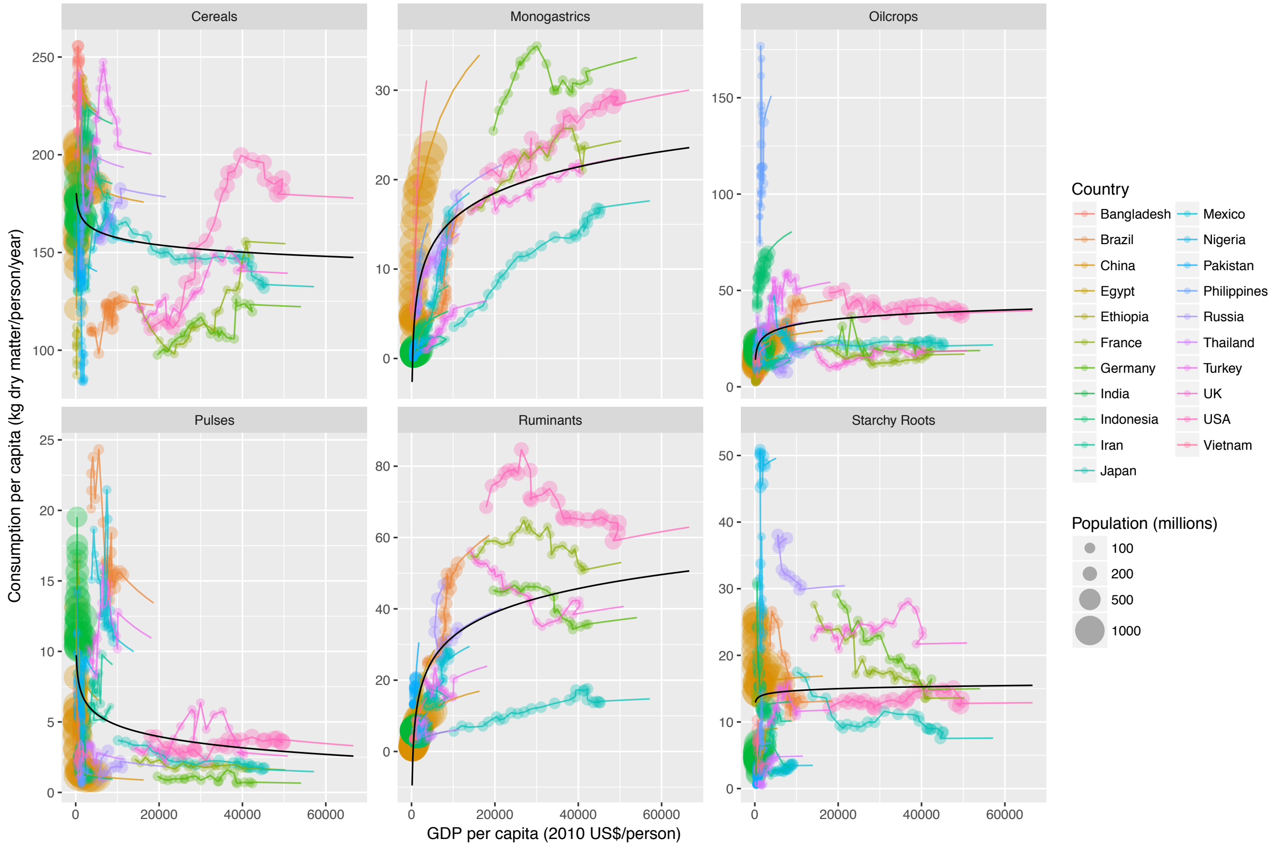

Figure SI-3. Per capita commodity group consumption by GDP for countries with a population greater than 60 million in 2010, as well as best fit log-linear curves and R^2 values from full country dataset. Coloured lines showing historical data 1961-2010, with data points size proportional to population, black line show the fitted log-linear curve, and projected data to 2030 shown as extended lines for each country.

Country level land use & international trade optimisation model

<u>Indices</u>

i	spatial locations within a country
j	crop and pasture land use types (wheat, maize, rice, oilcrops, pulses, starchy roots, energy crops and pasture)
j-crops	crop land use types, i.e. excluding pasture.
k	all agricultural commodities, including animal products
k-noncereals	agricultural crop commodities excluding cereals, i.e. oilcrops, pulses, starchy roots and energy crops
k-cereals	agricultural cereal commodities, i.e. wheat, maize and rice

k-feeds	crops for use as livestock feed, i.e. wheat, maize and oilcrops
k-feeds_and_pasture	feed crops plus pasture
l	land cover types (cropland, pasture and natural)

<u>Variables</u>

$area_{i,,j}$	area for crop *j* at location *i* (ha)
$f_{i,,j}$	fertiliser intensity factor for crop *j* at location *i* (unitless)
$w_{i,,j}$	irrigation intensity factor for crop *j* at location *i* (unitless)
$m_{i,,j}$	management intensity factor for crop *j* at location *i* (unitless)
$ruminant\_feed_k$	ruminant feed use for commodity *k* (t)
$monogastric\_feed_k$	monogastric feed use for commodity *k* (t)
$import_k$	imports for commodity *k* (t)
$export_k$	exports for commodity *k* (t)

All variables are positive. The intensity values (*f*, *w* and *m*) are indexes in range 0 to 1.

<u>Parameters</u>

$demand_k$	demand for commodity *k* (t)
$export\_pc_k$	export price for commodity *k* ($/t)
$import\_pc_k$	import price for commodity *k* ($/t)
$base\_lu\_cost_j$	base land use costs for crop *j* ($/ha)
f_cost	cost per tonne of fertiliser ($/t)
$w\_cost\_index_i$	irrigation cost index at location *i* (unitless, 0-1 range)
w_cost	irrigation cost ($/m^2)
m_cost_j	management intensity cost for crop *j* ($)
f_max	maximum fertiliser rate, i.e. at *f*=1(t/ha)
$w\_max_{i,j}$	maximum irrigation rate, i.e. at *w*=1, for crop *j* at location *i* (m^2/ha)
irrigiation_eff	Irrigation efficiency (%)
$crop\_dm_k$	crop dry matter for commodity *k* (%)

$suitable\_area_i$ area suitable for agriculture at location i (ha)

$water\_avail_i$ water availability at location i (m^3/ha)

$min/max\_net\_import_k$ minimum and maximum net imports for commodity k (t)

$lc\_change\_unit\_cost_l$ land cover change unit cost for cover type l (\$/ha)

All quantities (e.g. demand and trade amounts) for animal products were represented in dry matter feed equivalents based on feed conversion ratios (Alexander *et al.*, 2016).

<u>Constraints and calculations</u>

Unit land use costs and productivity

$$lu\_unit\_cost_{i,j} = base\_lu\_cost_j + f\_cost_j * f_{i,j} * f\_max + m\_cost_j * m_{i,j}$$
$$+ w\_cost * w\_cost\_index_i * w_{i,j} * w\_max_{i,j} * irrigiation\_eff$$

$yield_{i,j}$ calculated as described above.

Demand constraints

$$net\_supply_k = \sum_i yield_{i,j} * area_{i,j} - feed_k + import_k - export_k$$

$$demand_{k-noncereal\_crops} \geq net\_supply_{k-noncereal\_crops}$$

$$demand_{k-cereals} \geq \sum_{k-cereals} net\_supply_k$$

$$\sum_{k-feeds\_and\_pasture} ruminant\_feed_k * crop\_dm_k$$
$$\leq demand_{rumiants} - import_{ruminant} - export_{ruminants}$$

$$\sum_{k-feeds} monogastric\_feed_k * crop\_dm_k$$
$$\leq demand_{monogastrics} - import_{monogastrics} - export_{monogastrics}$$

International import/export constraints

$$import_k - export_k \leq max\_net\_import_k$$

$$import_k - export_k \geq min\_net\_import_k$$

$$import_{pasture} = 0$$

$$export_{pasture} = 0$$

Irrigation water availability constraint

$$water\_avail_i * suitable\_area_i \geq \sum_j w\_max_{i,j} * \ w_{i,j} * area_{i,j}$$

Land area constraints

$$suitable\_area_i \geq \sum_{j-crops} area_{i,j} + area_{i,pasture}$$

Land cover area changes

$$lc\_change_{natural} = abs\left(\sum_j (area_{i,j} \ - prev\_area_{i,j})\right)$$

$$lc\_change_{crop} = abs\left(\sum_{j-crops} (area_{i,j} \ - prev\_area_{i,j})\right)$$

$$lc\_change_{pasture} = abs(area_{i,pasture} - prev\_area_{i,pasture})$$

<u>Objective function</u>

The objective function of the optimisation was to minimise total country cost, given by:

$$Total\ country\ cost\ = \sum_i \sum_j cost\ area_{i,j} * lu_{unit_{cost}\,i,j}$$

$$+ \sum_i \sum_l lc\_change_{i,l} * lc\_change\_unit\_cost_l$$

$$+ \sum_k import_k * import\_pc_k - \sum_k export_k * export\_pc_k$$

International market price adjustment

The international export price $export\_pc_{k,t}$, of a commodity *k* at time *t*, is given by:

$$export\_pc_{k,t} = export\_pc_{k,t-1}\, e^{\frac{z_{k,t-1}}{\lambda}}$$

where $export\_pc_{k,t-1}$ is the price at time *t-1*, and $z_{k,t-1}$ is the excess demand normalised by the number of market participants at time *t-1*. λ is the parameter controlling the rate of market adjustment to market signals, and $z_{k,t}$ is given by:

$$z_{k,t} = \frac{global\_imports_{k,t} - global\_exports_{k,t}}{\max\,(global\_import_{k,t}, \text{global\_export}_{k,t})}$$

where $global\_imports_{k,t}$ is the global sum of imports of all countries at time *t* for commodity *k*. $global\_exports_{k,t}$ is the sum of global exports after transport losses at a rate of *t_loss*, representing the quantity available for import. The global import and export values are calculated as:

$$global\_imports_{k,t} = \sum_{countries} import_{k,t}$$

$$global\_exports_{k,t} = (1 - t\_loss) * \sum_{countries} export_{k,t}$$

The market sensitivity parameter, λ, was chosen as 0.3, in line with other models taking a similar dynamic non-equilibrium market approach (Ghoulmie *et al.*, 2005; Alexander *et al.*, 2013).

The import price $import\_pc_{k,t}$, is calculated from the international export price to account for transport costs t_cost, transport losses *t_loss*, and the import tariffs *i_tariff*, as:

$$import\_pc_{k,t} = export\_pc_{k,t} * \frac{(1 + i\_tariff)}{(1 - t\_loss)} + t\_cost$$

PLUMv2 parameters

Table SI-2. Crop cost and dry matter parameters

Crop type	Base cost ($/ha)	Max. management intensity cost ($/ha)	Dry Mass (%)
Wheat	112	508	0.87
Maize	91	469	0.86
Rice	140	560	0.89
Oilcrops	84	456	0.88
Pulses	109	502	0.31
Starchy roots	1624	3316	0.21
Miscanthus	119	521	*
Pasture	30	800	*
Note: * Miscanthus and pasture are represented in dry mass quantities.			

Table SI-3. PLUMv2 model parameters used. A uniform distribution was sampled across a range 50% either side of the central values.

Parameter	Central value
Irrigation cost, *w_cost* ($/m^2)	0.5
Fertiliser cost, *f_cost* ($/t)	1800
Other intensity cost, *m_cost* ($ at max management input)	600
Land cover change cost, *lc_change*: Natural to agricultural ($/ha)	60
Land cover change cost, *lc_change*: Managed forest to agricultural ($/ha)	160
Land cover change cost, *lc_change*: Agricultural land to natural ($/ha)	200
Land cover change cost, *lc_change*: Pasture to cropland ($/ha)	220
Land cover change cost, *lc_change*: Cropland to pasture ($/ha)	370
Minimum natural or managed forest cover	10%
Pasture harvest fraction	50%
Seed and waste rate	10%
Technology yield change rate, δ, above that from intensification of production	0.2%
Initial price shift factor	1.0
International market price sensitivity, λ	0.3
International import tariff, *i_tariff*	20%
Transport costs, *t_cost* ($/t)	50
Transportation losses, *t_loss*	5%

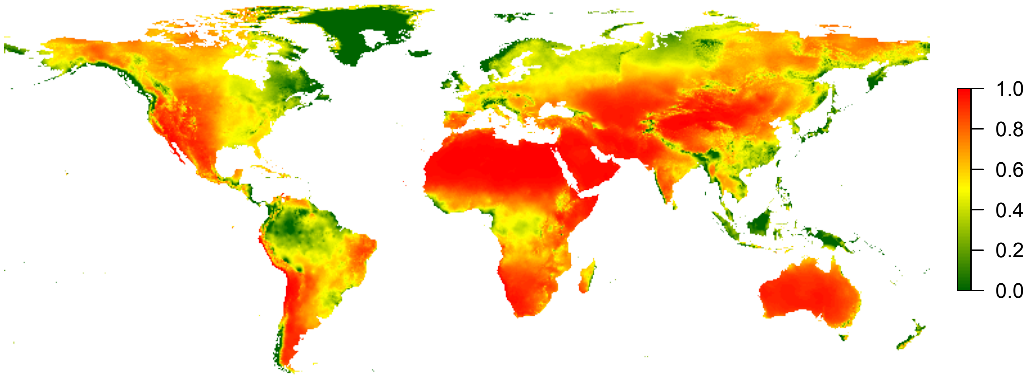

*Figure SI-4. Irrigation cost index (*w_cost_index*) used to determine unit irrigation water cost, derived from an aridity index (CGIAR-CSI, 2008).*

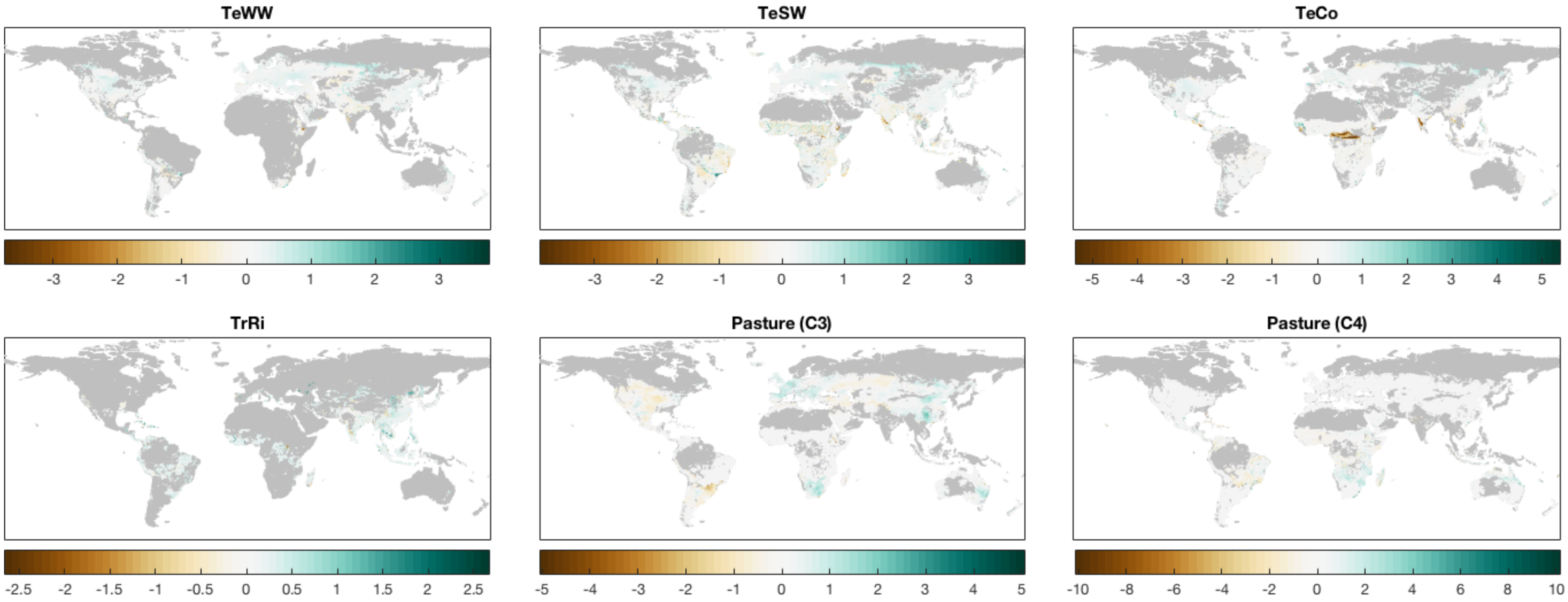

Figure SI-5. Changes in LPJ-GUESS yield potential (or, for C3 and C4 pasture grasses, annual net primary productivity) between 2011–2015 and 2096–2100 in tons/ha under RCP8.5. Crops were fully irrigated and received 200 kg/ha of nitrogen fertiliser per year.

1 References

Ahlström A, Schurgers G, Arneth A, Smith B (2012) Robustness and uncertainty in terrestrial ecosystem carbon response to CMIP5 climate change projections. *Environmental Research Letters*, **7**, 44008.

Alexander P, Moran D, Rounsevell MDA, Smith P (2013) Modelling the perennial energy crop market: the role of spatial diffusion. *Journal of the Royal Society Interface*, **10**.

Alexander P, Brown C, Rounsevell M, Finnigan J, Arneth A (2016) Human appropriation of land for food: The role of diet. *Global Environmental Change*, **41**, 88–98.

Allen RG, Pereira LS, Raes D, Raes D (1998) Chapter 8 - ETc under soil water stress conditions. In: *Crop evapotranspiration - Guidelines for computing crop water requirements*. Food and Agriculture Organization of the United Nations (FAO), Rome, Italy.

AQUASTAT (2016) *AQUASTAT database*. Food and Agriculture Organization of the United Nations (FAO), Rome, Italy.

CGIAR-CSI (2008) *Global Aridity and PET Database*.

Elliott J, Müller C, Deryng D et al. (2015) The Global Gridded Crop Model Intercomparison: Data and modeling protocols for Phase 1 (v1.0). *Geoscientific Model Development*, **8**, 261–277.

FAOSTAT (2015) *Food Supply - Crops Primary Equivalent (2015-12-16)*. Food and Agriculture Organization of the United Nations, Rome, Italy.

Ghoulmie F, Cont R, Nadal J-P (2005) Heterogeneity and feedback in an agent-based market model. *Journal of Physics: Condensed Matter*, **17**, S1259–S1268.

Harris I, Jones PD, Osborn TJ, Lister DH (2014) Updated high-resolution grids of monthly climatic observations - the CRU TS3.10 Dataset. *International Journal of Climatology*, **34**, 623–642.

Hurtt G, Chini L, Frolking S, Sahajpal R (2017) Land-Use Harmonization (LUH2). *Global Ecology Laboratory, University of Maryland*.

Portmann FT, Siebert S, Döll P (2010) MIRCA2000—Global monthly irrigated and rainfed crop areas around the year 2000: A new high-resolution data set for agricultural and hydrological modeling. *Global Biogeochemical Cycles*, **24**, 1–24.

Le Quéré C, Andrew RM, Canadell JG et al. (2016) Global Carbon Budget 2016. *Earth System Science Data*, **8**, 605–649.

Sitch S, Smith B, Prentice IC et al. (2003) Evaluation of ecosystem dynamics, plant geography and terrestrial carbon cycling in the LPJ dynamic global vegetation model. *Global Change Biology*, **9**, 161–185.

Taylor KE, Stouffer RJ, Meehl G a. (2012) An Overview of CMIP5 and the Experiment Design. *Bulletin of the American Meteorological Society*, **93**, 485–498.
